# Estradiol analogs attenuate autophagy, cell migration and invasion by direct and selective inhibition of TRPML1, independent of estrogen receptors

**DOI:** 10.1038/s41598-021-87817-4

**Published:** 2021-04-15

**Authors:** Philipp Rühl, Anna Scotto Rosato, Nicole Urban, Susanne Gerndt, Rachel Tang, Carla Abrahamian, Charlotte Leser, Jiansong Sheng, Archana Jha, Günter Vollmer, Michael Schaefer, Franz Bracher, Christian Grimm

**Affiliations:** 1 Department of Pharmacy – Center for Drug Research, Ludwig-Maximilians University, Munich, Germany; 2 Walther Straub Institute of Pharmacology and Toxicology, Faculty of Medicine, Ludwig-Maximilians University, Munich, Germany; 3 Rudolf-Boehm-Institute for Pharmacology and Toxicology, University of Leipzig, Leipzig, Germany; 4 CiPA LAB, LLC, Gaitherburg, MD USA; 5 Casma Therapeutics Inc, Cambridge, MA USA; 6 Institute of Zoology, Molecular Cell Physiology and Endocrinology, University of Dresden, Dresden, Germany

**Keywords:** Biophysical chemistry, Oncology, Chemistry, Chemical biology

## Abstract

The cation channel TRPML1 is an important regulator of lysosomal function and autophagy. Loss of TRPML1 is associated with neurodegeneration and lysosomal storage disease, while temporary inhibition of this ion channel has been proposed to be beneficial in cancer therapy. Currently available TRPML1 channel inhibitors are not TRPML isoform selective and block at least two of the three human isoforms. We have now identified the first highly potent and isoform-selective TRPML1 antagonist, the steroid 17β-estradiol methyl ether (EDME). Two analogs of EDME, PRU-10 and PRU-12, characterized by their reduced activity at the estrogen receptor, have been identified through systematic chemical modification of the lead structure. EDME and its analogs, besides being promising new small molecule tool compounds for the investigation of TRPML1, selectively affect key features of TRPML1 function: autophagy induction and transcription factor EB (TFEB) translocation. In addition, they act as inhibitors of triple-negative breast cancer cell migration and invasion.

## Introduction

TRPML1 is a lysosomal cation channel of the transient receptor potential (TRP) superfamily permeable for Ca^2+^, Na^+^, Fe^2+^, Zn^2+^, and other cations. TRPML1 is involved in a number of physiological processes and human diseases. Loss or mutation of TRPML1 causes the neurodegenerative lysosomal storage disorder mucolipidosis type IV^1^; vice versa activation of TRPML1 clears intraneuronal Aβ in preclinical models of HIV infection^2^ and protects human dopaminergic neurons from α-synuclein toxicity through increased lysosomal exocytosis^3^. TRPML1 is also essential for sarcolemma repair to prevent muscular dystrophy^4^, and small molecule activation of TRPML1 ameliorates Duchenne muscular dystrophy^5^. TRPML1 further plays a role in gastric acid secretion and may represent a therapeutic target for chronic Helicobacter pylori infection^6–8^. TRPML1 also acts as ROS (reactive oxygen species) sensor in lysosomes^9^, it regulates autophagy through calcineurin and transcription factor EB (TFEB)^10^ or in a TFEB-independent manner^11,12^, it regulates lysosomal motility and lysosomal positioning^13^, and it plays a role in osteoclastogenesis and bone remodeling^14^. Furthermore, TRPML1 has functions in the immune system, e.g. it controls the migration of dendritic cells^15^ and it plays a role in the education process of natural killer (NK) cells^16^. Finally, HRAS-driven cancer cells, i.e. cells containing mutations in HRAS, a small GTPase of the Ras superfamily, are vulnerable to TRPML1 inhibition^17^. Loss of TRPML1 impairs the growth of melanoma^18^ and reduces cell proliferation, cell viability and tumor growth in the MDA-MB-231 breast cancer cell line, a highly aggressive, invasive and poorly differentiated triple-negative breast cancer (TNBC) cell line lacking estrogen and progesterone receptor expression as well as HER2 (human epidermal growth factor receptor 2) amplification^18^.

In recent years several TRPML channel activators have been developed by different groups, e.g. SF-22, MK6-83, ML-SA1, or ML-SA5^5,19–21^. In addition, TRPML isoform-selective agonists have been discovered recently such as a TRPML2-selective agonist, ML2-SA1^22^ or TRPML3-selective agonists, SN-2 and EVP-21^20,22^. TRPML1-selective agonists are currently not available, neither are TRPML1-selective antagonists. Only inhibitors without isoform-selectivity, e.g. ML-SI1 and ML-SI3 have been described so far^23,24^. TRPML channel isoforms can occur not only in the same cell type, e.g. in certain types of macrophages and other immune cells, but also in the same type of organelle, e.g. in early endosomes (EE), late endosomes (LE), or lysosomes (LY). In particular, TRPML1, 2, and 3 in LE/LY and TRPML2 and 3 in EE. Isoform-selective agonists and antagonists are therefore highly desired as chemical tools to decipher channel functions in endogenously expressing cell systems and organelles with TRPML isoform-heterogeneity; and therapeutically to exclude potential side effects mediated by inhibition or activation of the other TRPML isoforms. We have screened a library of 2430 drug-like small molecule compounds, the majority of which from the Spectrum Collection compound library (MS Discoveries; 2000 compounds) containing numerous FDA-approved drugs, to identify TRPML isoform-selective inhibitors. In the following we describe the first highly potent and subtype-selective antagonist of TRPML1, 17β-estradiol methyl ether (EDME), which we further modified chemically to improve its characteristics. Thus, analogs of EDME were generated with reduced estrogen receptor alpha (ERα) activity. Functionally, we found that EDME and its analogs inhibit autophagy and translocation of TFEB, a master regulator of autophagy and lysosomal biogenesis, to the nucleus by direct and selective inhibition of TRPML1. In human estrogen receptor negative (ER-) breast cancer cells (MDA-MB-231) EDME was found to reduce migration and invasion, corroborating the efficacy of these compounds to interfere with cancer hallmarks in a TRPML1-dependent but estrogen receptor independent manner.

## Results

### Identification of EDME as selective TRPML1 antagonist

**Figure 1:**
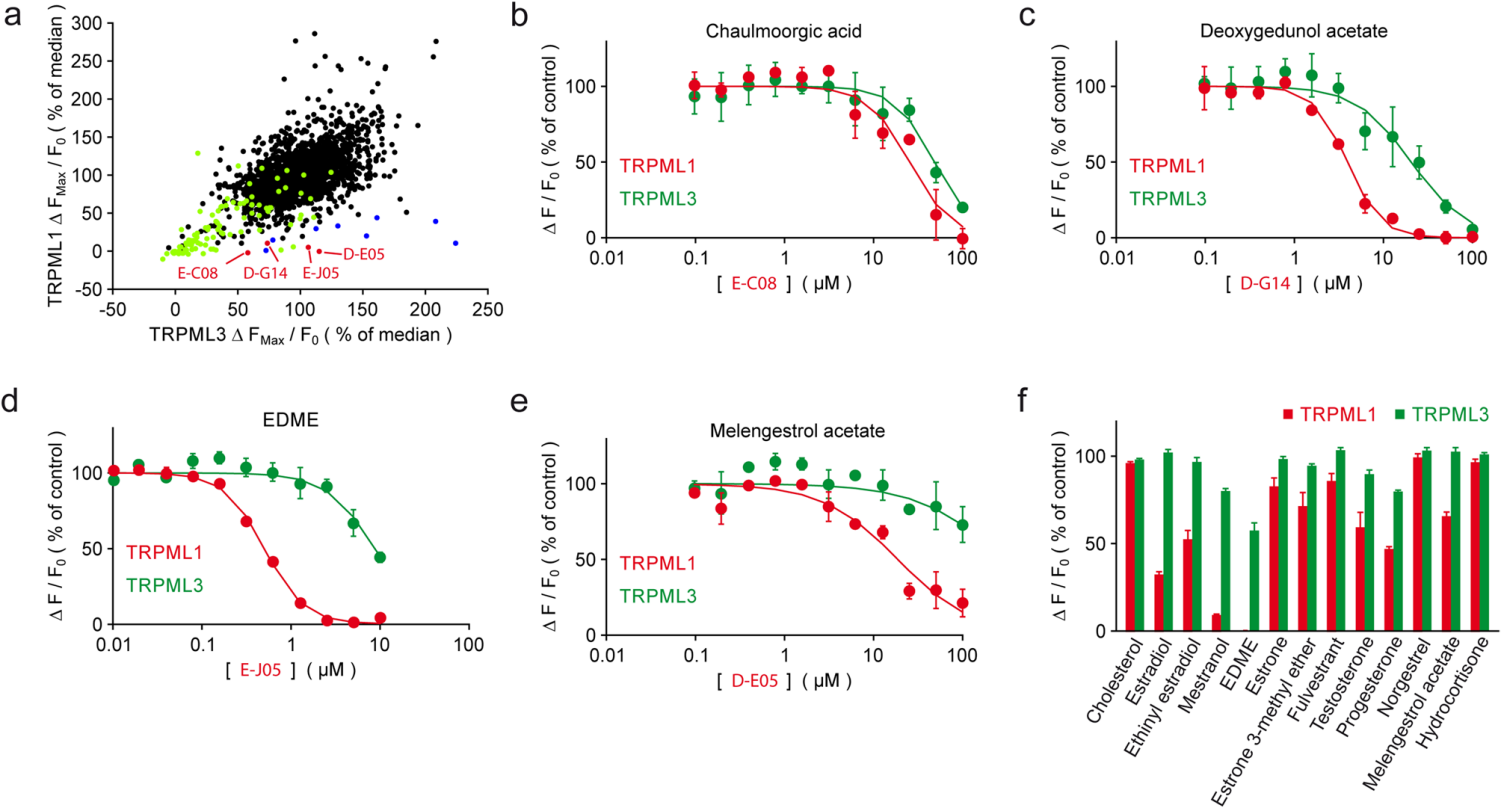
Compound screening and hit validation. (**a**) Dot plot showing the inhibitory effects of 2430 bioactive compounds (20 µM) on fluo4-loaded HEK293 cell lines stably expressing hTRPML1∆NC-YFP and hTRPML3-YFP after activation of the cells with 5 µM ML-SA1. Peak fluorescence intensities after stimulation were normalized to the median response in the respective screening plate. Black dots indicate compounds with no discernible effect on either TRPML1 or TRPML3. Green dots represent fluorescent compounds that were not further considered. Blue dots are notoriously positive hits previously identified in several other screened targets. Red dots indicate TRPML1-selective hits. (**b**–**e**) Representative concentration–response curves of the 4 specific hits in (A) for TRPML1 (red dots and lines) and TRPML3 (green dots and lines). (**f**) Effect of retested and pharmacologically relevant steroids on TRPML1 and TRPML3 at a concentration of 12.5 µM, which in the case of EDME completely blocks TRPML1. Shown is the inhibition of Fluo-4 fluorescence responses to stimulation with 5 µM ML-SA1 and normalization to an untreated control. Aggregated data sets from 3 to 5 independent experiments, each performed in duplicates, are displayed (means ± SEM).

**Figure 2:**
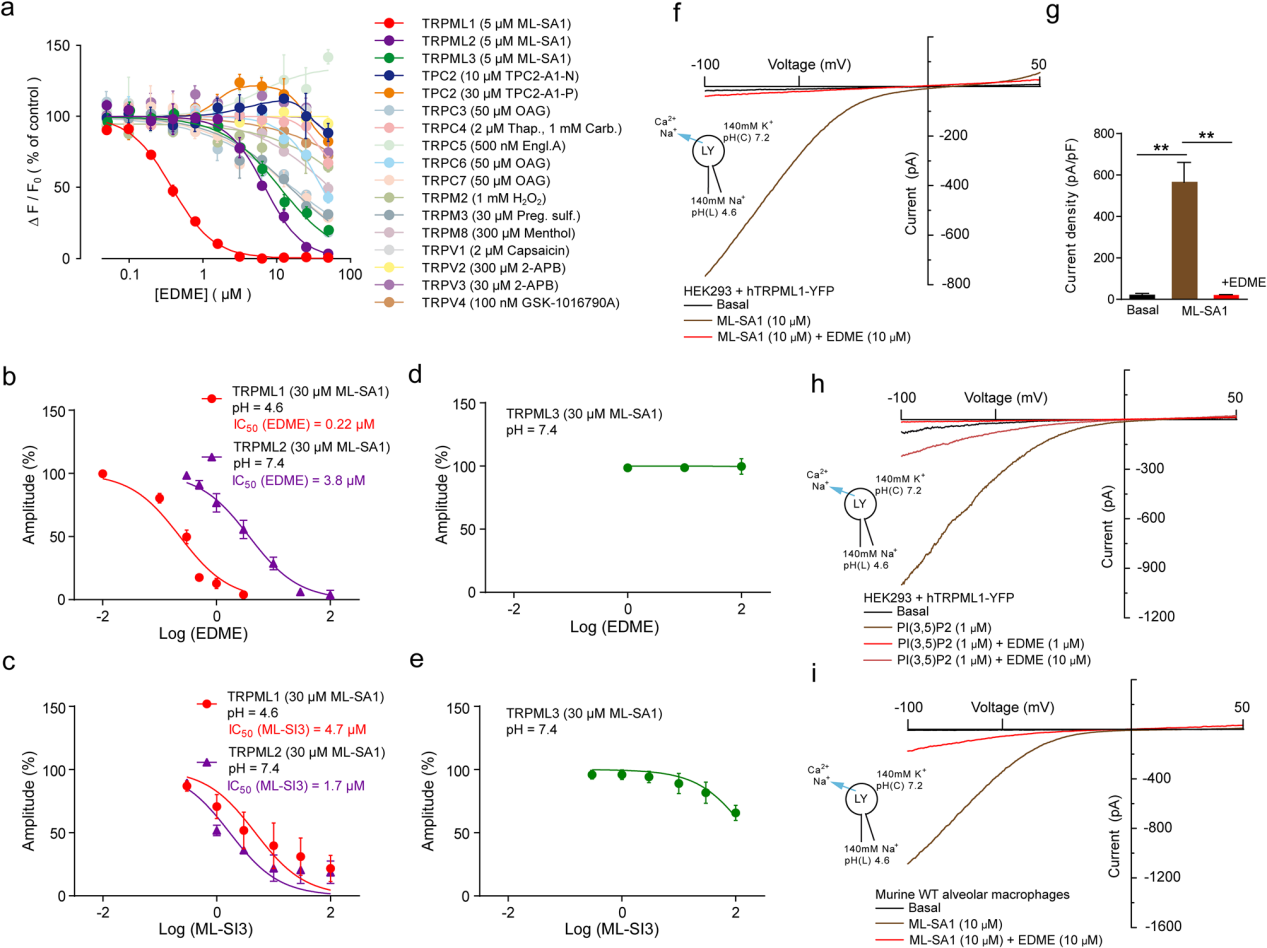
EDME is a potent and selective blocker of TRPML1. (**a**) Representative concentration-effect relationships for Ca^2+^ increases (Fluo-4) in response to different concentrations of EDME on HEK293 cells stably expressing different TRP channels including hTRPML1∆NC, 2, 3, or TPC2, respectively. Corresponding activators for each TRP channel are listed in parentheses. (**b**–**e**) Concentration-effect relationships obtained from whole-cell patch-clamp measurements showing effect of EDME (**b**, **d**) and the previously described non-selective TRPML blocker ML-SI3 (**c**, **e**) on hTRPML1L15/16A, L577/578A (pH 4.6; n = 6, each), hTRPML2 (pH 7.4; n = 4, each), and hTRPML3 (pH 7.4; n = 3, each) in the presence of 30 µM ML-SA1. Current recording was done with WinWCP5.2.7 (University of Strathclyde, UK) software, and analysis was done with the help of a customized Igor pro program (WaveMetrics). (**f**) Endolysosomal patch-clamp experiment showing effect of EDME on WT hTRPML1 after activation with ML-SA1 (10 µM). (**g**) Statistical analysis for experiments as shown in (**f**) (luminal pH 4.6; n = 3, each). P-values were calculated by one-way ANOVA followed by Tukey’s post hoc test. **p-value < 0.01. (**h**) Endolysosomal patch-clamp experiment showing effect of EDME on WT hTRPML1 after activation with the endogenous ligand PI(3,5)P_2_ (1 µM). (**i**) Endolysosomal patch-clamp experiment showing effect of EDME (10 µM) on TRPML1 endogenously expressed in mouse alveolar macrophages after activation with ML-SA1 (10 µM). All endolysosomal patch-clamp experiments were analyzed using PatchMaster acquisition software (https://www.heka.com/) and OriginPro 6.1 (https://www.originlab.com/). All statistical analysis was done using GraphPadPrism software (https://www.graphpad.com/scientific-software/prism/).

Initially, 2430 compounds were tested on hTRPML1∆NC-YFP, a plasma membrane variant of wild-type TRPML1 lacking N- and C-terminal lysosomal targeting sequences as reported previously^22^, and hTRPML3-YFP, both stably expressed in HEK293 cells. TRPML1 and TRPML3 were activated with 5 µM ML-SA1, respectively (Fig. 1a), a concentration which showed no effect in the parental control cell line. Four TRPML1 selective hit compounds [the synthetic gestagen melengestrol acetate (D-E05; MGA), 17β-estradiol methyl ether (E-J05; EDME), the natural triterpene deoxygedunol acetate (D-G14; DGA), and the fatty acid derivative chaulmoorgic acid (E-C08)] were initially identified and subsequently retested by performing concentration–response measurements (Fig. 1b–e, S1). MGA and EDME were thus confirmed as highly TRPML1-selective with IC_50_ values of 18.6 µM (MGA) and 0.5 µM (EDME). EDME showed the lowest IC_50_ for TRPML1. Subsequently, other pharmacologically relevant steroidal compounds were tested. Of those, estradiol, ethinylestradiol, and mestranol which is the 3-methyl ether of ethinylestradiol also showed stronger inhibitory effects on TRPML1 than on TRPML3 with mestranol showing the strongest effect. However, none of these reached the TRPML1-inhibitory activity of EDME. Other steroid hormones like progesterone and testosterone showed much weaker inhibitory effects compared to EDME or mestranol while cholesterol, hydrocortisone, estrone, estrone 3-methyl ether, norgestrel, and fulvestrant, the latter one used to treat hormone receptor positive metastatic breast cancer, had no effect on either TRPML1 or TRPML3 (Figs. 1f, S1). TRPML channels belong to the superfamily of transient receptor potential (TRP) channels. Those are the channels TRPMLs are most closely related with. Hence, we have counter screened EDME against a plethora of other members of the TRP superfamily, namely TRPC3, 4, 5, 6, and 7 of the canonical or classical TRP subfamily, members of the melastatin subfamily, TRPM2, 3, and 8 as well as members of the vanilloid subfamily, TRPV1, 2, 3, and 4. We further repeated testing against TRPML2 and TRPML3 as well as the functionally related endolysosomal two-pore cation channel TPC2. Channels were activated with previously reported activation concentrations of known ligands, respectively (Fig. 2a). While no meaningful IC_50_s were obtained for EDME for most channels including TPC2, activated by either TPC2-A1-P or TPC2-A1-N as reported recently^25^, the following IC_50_s were determined for TRPML1, 2 and 3: 0.6 µM, 5.9 µM, and 19.5 µM (Table S1). To further corroborate these data we performed whole-cell patch-clamp experiments with EDME using stable cell lines expressing either the plasma membrane variant hTRPML1^L15/16A, L577/578A^, hTRPML2, or hTRPML3. While no block for TRPML3 was found, TRPML2 was blocked with an IC_50_ of 3.8 µM. The IC_50_ measured for TRPML1 was 0.22 µM (Fig. 2b,d). For comparison, we also tested the recently described TRPML inhibitor ML-SI3 which blocked TRPML1 with an IC_50_ of 4.7 µM and TRPML2 with an IC_50_ of 1.7 µM, suggesting that ML-SI3 has an almost threefold stronger effect on TRPML2 compared to TRPML1 and is > 20-fold weaker on TRPML1 than EDME (Fig. 2c,e). Next, we confirmed the inhibitory effect of EDME in endolysosomal patch-clamp experiments by isolating vacuolin-enlarged endo-lysosomes from hTRPML1 WT expressing HEK293 cells (Fig. 2f–h). Finally, we used TRPML1 endogenously expressing murine alveolar macrophages to confirm inhibition of TRPML1 activation by EDME (Fig. 2i).

### Systematic modification of EDME

**Figure 3:**
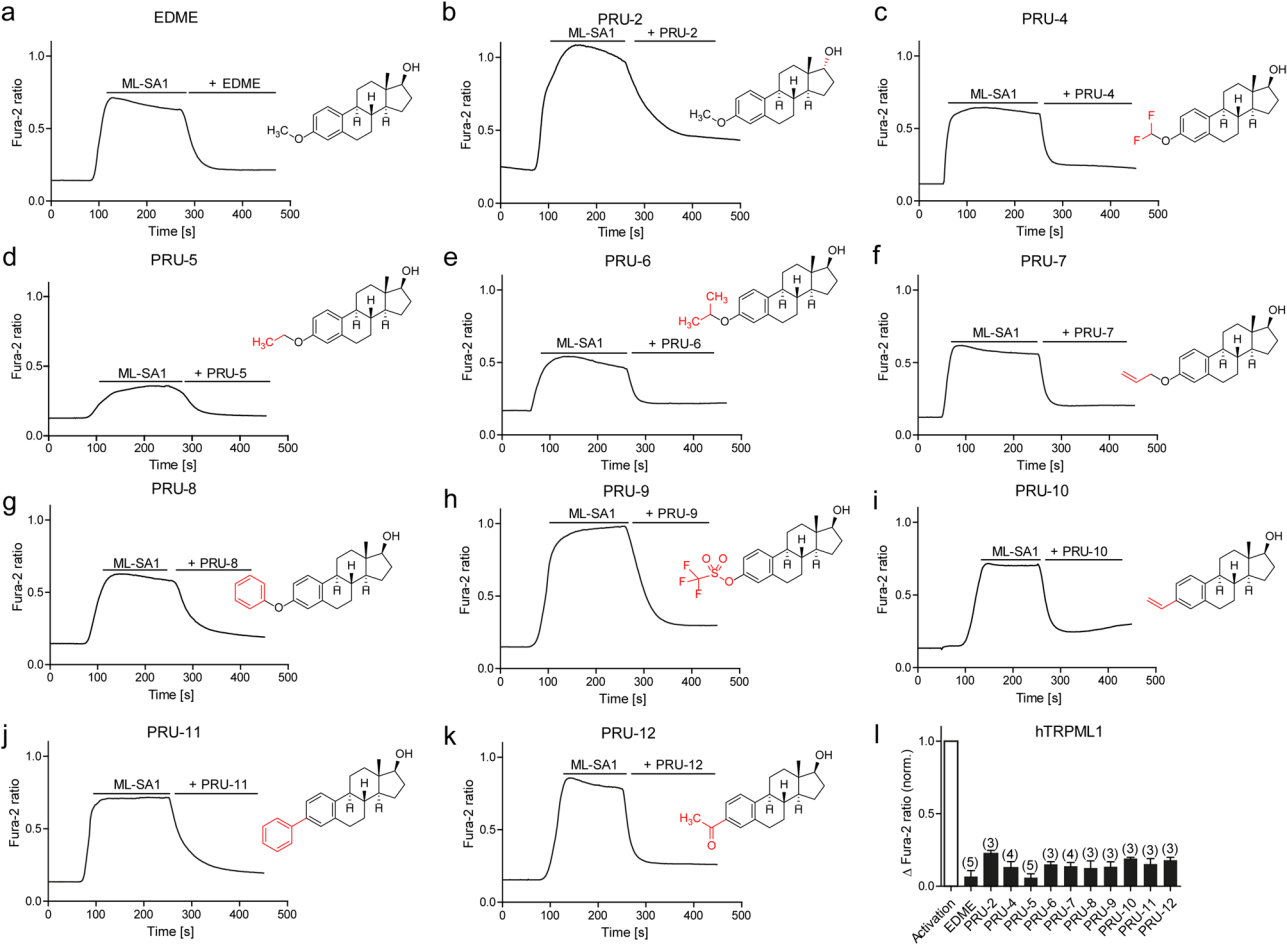
EDME and analogs in Fura-2 single cell calcium imaging experiments. (**a**–**k**) Representative Fura-2 calcium signals recorded from HEK293 cells stably expressing hTRPML1∆NC-YFP. Cells were stimulated with ML-SA1 (10 µM), then treated with EDME and analogs (10 µM, each). Characteristic structural motifs of the EDME analogs are highlighted in red in the structures. (**l**) Statistical analysis of the maximal change in Fura-2 ratio (mean ± SEM) with the number of independent experiments in parentheses (with 5–12 cells in each experiment). Data were normalized to the maximal effect of the agonist ML-SA1.

**Figure 4:**
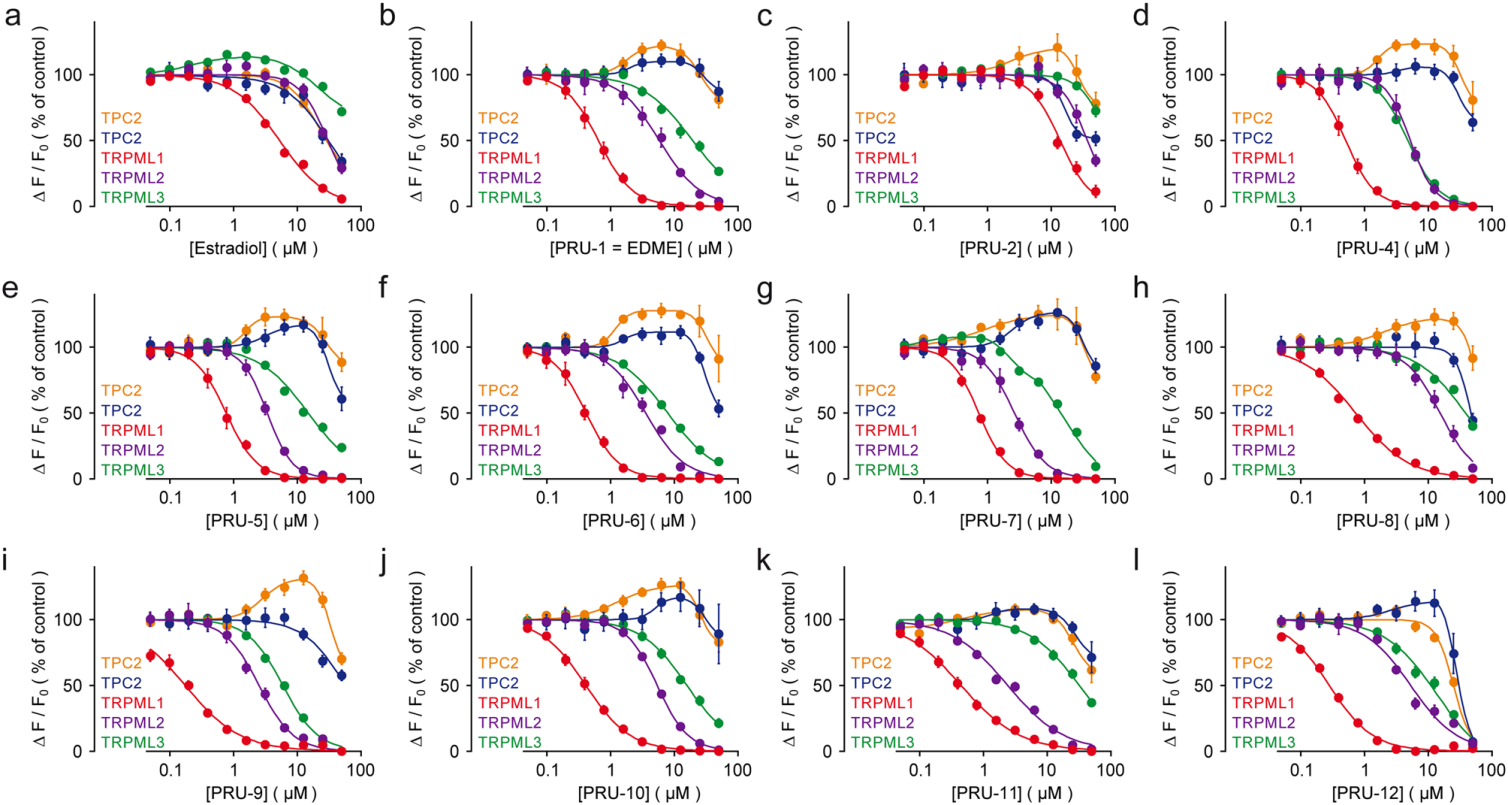
Estradiol, EDME and analogs in Fluo-4 calcium imaging experiments. (**a**–**l**) Concentration-effect relationships for Ca^2+^ increases (Fluo-4) in response to different concentrations of EDME, estradiol and analogs on HEK293 cells stably expressing hTRPML1∆NC-YFP, hTRPML2-YFP, hTRPML3-YFP or hTPC2L11A/L12A-RFP^22,24^. Cells were activated with ML-SA1 (5 µM) for TRPMLs or TPC2-A1-N (10 µM; blue) and TPC2-A1-P (30 µM; orange) for hTPC2. Data are calculated from 3 to 5 independent experiments, each, and represented as means ± SEM. IC_50_ values are presented in Table S1.

Initial structure–activity relationships (SAR) were detected by analyzing the structures of the screening hits and other steroidal compounds tested in either the random screening or the consecutive experiments. From these data it was evident that natural and synthetic steroids lacking an aromatic ring A (typical for estrogens) have virtually no (cholesterol, phytosterols, glucocorticoids, mineralocorticoids, antiestrogens, antiandrogens, 5α-reductase inhibitors) or only weak TRPML1-inhibitory activity (some androgens and gestagens) (Fig. 1f). In the class of gestagens progesterone and melengestrol acetate (MGA) had shown modest TRPML1 inhibition (IC_50_ = 12 µM and 19 µM, respectively). Further, stilbene-type synthetic estrogens and plant phytoestrogens were inactive in the primary screen. In the class of estrogens, the native hormone 17β-estradiol was significantly weaker (IC_50_ = 5.3 µM) than EDME. Likewise, the synthetic 17-ethinyl derivative ethinylestradiol was only weakly active. Modification of 17β-estradiol at 3-OH with an ionic residue (estradiol-3-sulfate sodium salt) eliminated TRPML1-inhibitory activity. Only mestranol, a congener of EDME bearing an additional ethinyl group at C-17, showed considerable activity. From these structures of mainly weakly active or inactive steroidal and related compounds it was evident that there is a very steep structure–activity relationship: only estrane-type compounds are promising and variations at ring D are most likely critical; the most obvious position for further modifications was position 3 at the aromatic ring A. Therefore we synthesized 10 modified versions of EDME, most of which have in common a replacement of the methoxy group at C-3 with a lipophilic residue. One single variation was performed on ring D by conversion of alfatradiol, the 17α epimer of physiological 17β-estradiol, into its 3-methoxy derivative PRU-2 by simple O-methylation^26^. A couple of ethers of 17β-estradiol were obtained by various etherification protocols: known O-alkyl derivatives like ethyl ether PRU-5^27^, isopropyl ether PRU-6^28^, and allyl ether PRU-7^29^ were obtained under standard Williamson conditions. Since for these trivial alkyl ethers undesired oxidative O-dealkylation by CYP enzymes to give free 17β-estradiol cannot be excluded^25^, we prepared two estradiol ethers with presumably high metabolic stability. Difluoromethyl ether PRU-4^30^ was obtained from 17β-estradiol using Zafrani’s diethyl (bromodifluoromethyl)phosphonate reagent^31^, and phenyl ether PRU-8 was prepared by O-phenylation with benzyne generated from 2-(trimethylsilyl)phenyl trifluoromethanesulfonate with fluoride ions^32^. In order to obtain lipophilic 17β-estradiol analogs without a C,O-bond at C-3, for which metabolism into the parent hormone should be fully excluded, we converted the phenolic group into the O-triflate to give the useful building block PRU-9^33^. Suzuki–Miyaura cross-coupling of PRU-9 with phenylboronic acid gave the 3-phenylestrane PRU-11, Stille cross-coupling with tributyl(vinyl)tin under Pd(II) catalysis^34^ the 3-vinylestrane PRU-10. Methyl ketone PRU-12^34^ was obtained via Stille cross-coupling of PRU-9 with tributyl(1-ethoxyvinyl)tin, followed by aqueous hydrolysis of the formed enol ether (Scheme S1). These EDME analogs were tested for TRPML1 inhibition in single cell Fura-2 calcium imaging experiments at a concentration of 10 µM (Figs. 3a–l and S2). Surprisingly, in these experiments all compounds showed strong efficacy in blocking TRPML1 with the exception of 17α-epimer PRU-2. In a next series of experiments, we performed concentration–response measurements with 17β-estradiol (E2), EDME and the 10 analogs. For analysis of subtype selectivity, we further tested all compounds on TRPML1, 2, and 3 as well as the related endolysosomal cation channel TPC2 (Fig. 4a–l). Again, 17α-epimer PRU-2 showed only poor activity, whereas all other EDME analogues with a 17β hydroxyl group were identified as potent inhibitors of TRPML1 with IC_50_ values below 1 µM (Table S1). PRU-8, PRU-9, PRU-10, and PRU-12 showed a further improved selectivity profile compared to EDME (Fig. 4h–j,l).

### Effect of EDME and analogs at estrogen receptor alpha

**Figure 5:**
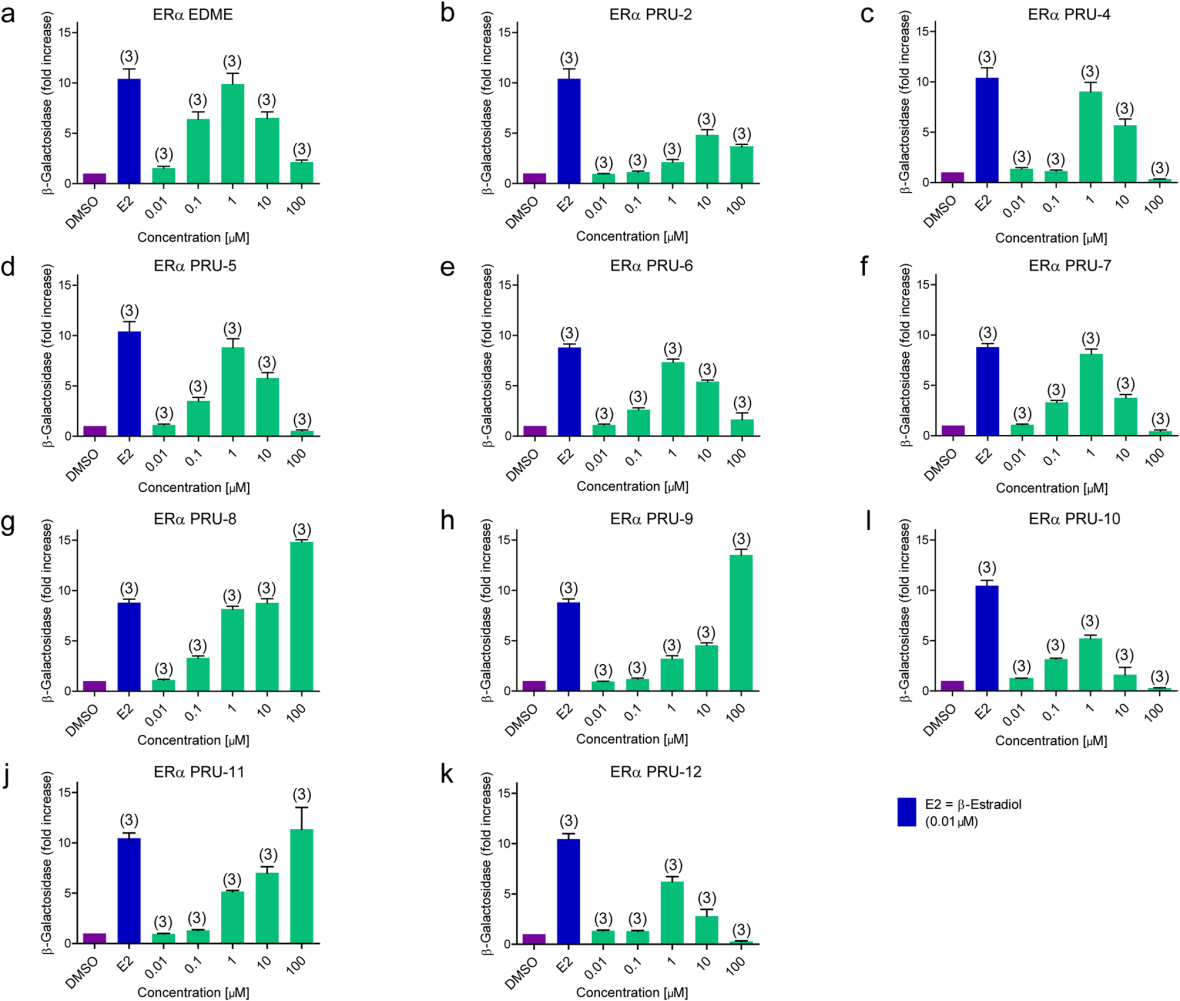
Activity at ERα of EDME and analogs. (**a**–**k**) Shown are effects of EDME and analogs at the estrogen receptor alpha (ERα) in *Saccharomyces cerevisiae* stably transfected with ERα, with the number of independent experiments in parentheses. The weakest effects at ERα were found for PRU-2, PRU-10, and PRU-12.

To assess the effect of EDME and its synthetic analogs at ERα, we performed a yeast estrogen receptor assay used to determine the relative transactivation activity of the human ERα in response to test substances as previously described^35^. Briefly, Saccharomyces cerevisiae stably transfected with a human ERα construct and an estrogen responsive element fused to the reporter gene lacZ encoding for β-galactosidase were treated with the test substances. We found that PRU-2, PRU-10 and PRU-12 had relatively low efficacy at ERα while EDME and the other analogs had comparably strong effects (Fig. 5). PRU-2, the 17α-epimer of EDME, showed the desired low efficacy at ERα, but since it had a much higher IC_50_ (14 µM) for TRPML1 compared to EDME this compound was not further considered. PRU-8 showed a very good selectivity profile for TRPMLs, but unfortunately a strong effect at ERα, whereas PRU-10 and PRU-12 showed both a low ERα activity and a good TRPML isoform selectivity profile. In the following experiments, we therefore focused on EDME, PRU-10, and PRU-12.

### Effect of EDME and selected analogs on autophagy and TFEB translocation

**Figure 6:**
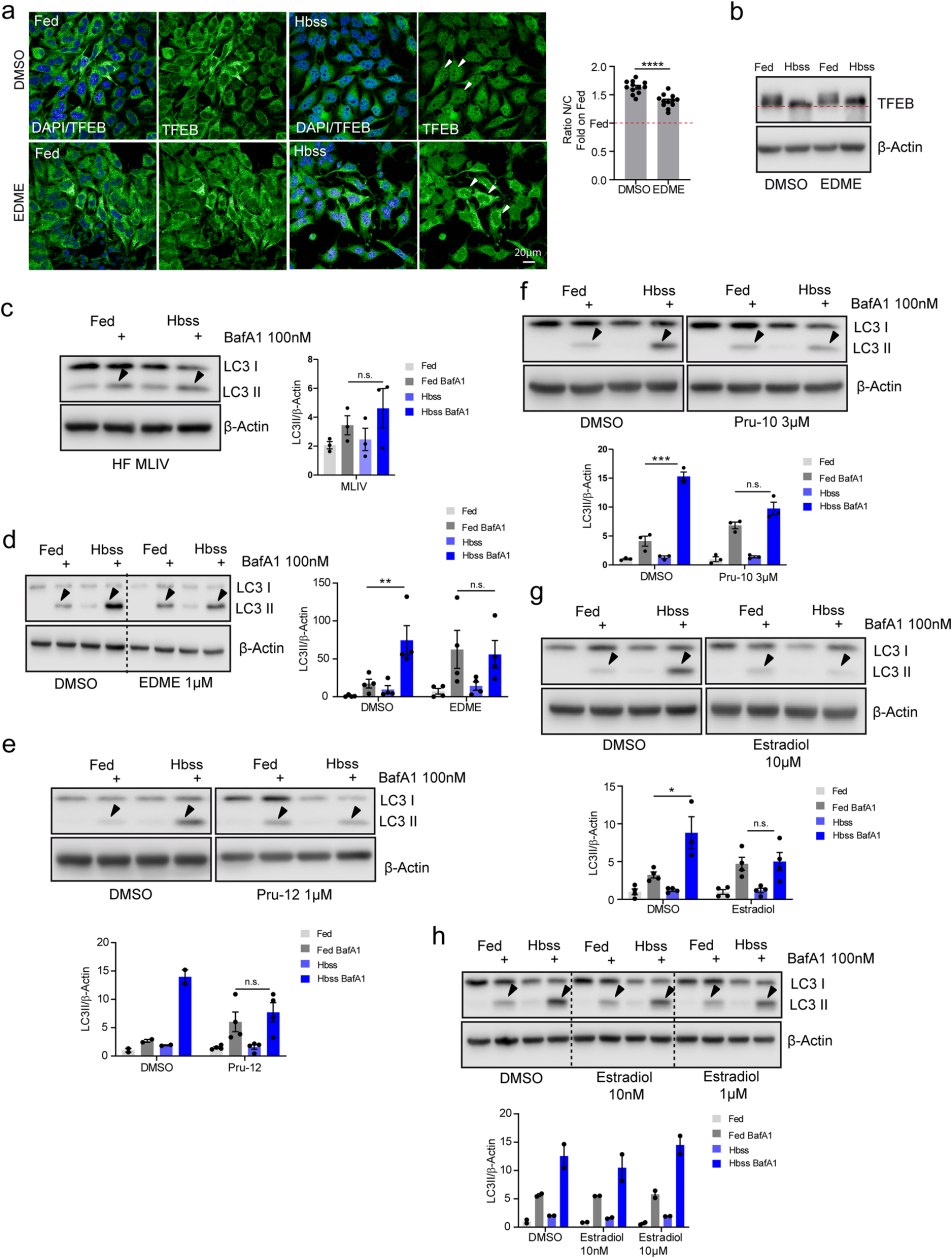
Effect of EDME and selected analogs on autophagy and TFEB nuclear translocation. (**a**) Representative confocal images of endogenous TFEB in HeLa cells treated with DMSO and EDME (1 µM) in complete media (Fed) or Hbss (nutrient starved media). The plot represents the TFEB nuclear to cytosol ratio and values are expressed as fold induction on Fed. Values are means ± SEM of n = 300 cells per condition, pooled from two independent experiments. (**b**) Representative image of immunoblot analysis of endogenous TFEB in human fibroblasts treated with DMSO and EDME (1 µM) in Fed and Hbss. The red dashed line highlights TFEB molecular downshift. (**c**) Representative image of immunoblot analysis of endogenous LC3 in MLIV patients’ fibroblasts treated with Fed and Hbss alone or in the presence of BafA1 (bafilomycin A1). Plot shows the densitometry of LC3II band normalized to actin. The data in the graphs are mean values ± SEM, n = 3 lysates per condition pooled from 3 independent experiments. (**d**–**h**) Representative image of immunoblot analysis of endogenous LC3 in human fibroblasts wild type treated with DMSO, EDME 1 µM, PRU-12 1 µM, PRU-10 3 µM and estradiol (10 µM, 1 µM, and 10 nM) in Fed and Hbss alone or in the presence of BafA1. Plot shows the densitometry of LC3II band normalized to actin. The data in the graphs are mean values ± SEM, n = 2–4 lysates per condition pooled from 2 to 4 independent experiments. P-values were calculated by two-tailed Student’s t-test. *p-value < 0.05; **p-value < 0.01. All statistical analysis was done using GraphPadPrism software (https://www.graphpad.com/scientific-software/prism/).

TRPML1 plays a major role in autophagy and starvation-stimulated TFEB nuclear translocation^10,11^. Indeed, cells from Mucolipidosis type IV (MLIV) patients show a delayed fusion of autophagosomes with late endosomes/lysosomes and alterations in TFEB shuttling^10,36,37^. In order to evaluate the efficacy of the novel compounds on TRPML1 functions we tested their effects on TFEB dephosphorylation and autophagic flux. As previously demonstrated for other, non-selective inhibitors, treatment with EDME reduced TFEB molecular downshift induced by nutrient starvation (Figs. 6a,b and S3). In order to evaluate lysosome-dependent degradation of autophagic material, we treated cells with EDME, PRU-10, and PRU-12 in combination with bafilomycin A1, a potent V-ATPase inhibitor^38^. Cells treated with TRPML1 inhibitors behaved similarly to MLIV patient fibroblasts which lack functional TRPML1 (Figs. 6c and S3). No increase in LC3 compared to vehicle (DMSO) was found, suggesting an impairment in the induction of autophagy upon starvation (Figs. 6d–f and S3). EDME and PRU-12 had the strongest effects, showing efficacy at as low as 1 µM. For PRU-10 a higher effective concentration was needed (3 µM). Estradiol showed effects when a concentration of 10 µM was applied. Concentrations of 10 nM or 1 µM showed no effect (Figs. 6g–h and S3). Taken together, these results demonstrate functional efficacy of EDME and analogs in blocking endogenous TRPML1 activity in intact cells, establishing them as suitable tools for cellular assays.

### Effect of EDME and selected analogs on breast cancer cell migration and invasion

**Figure 7:**
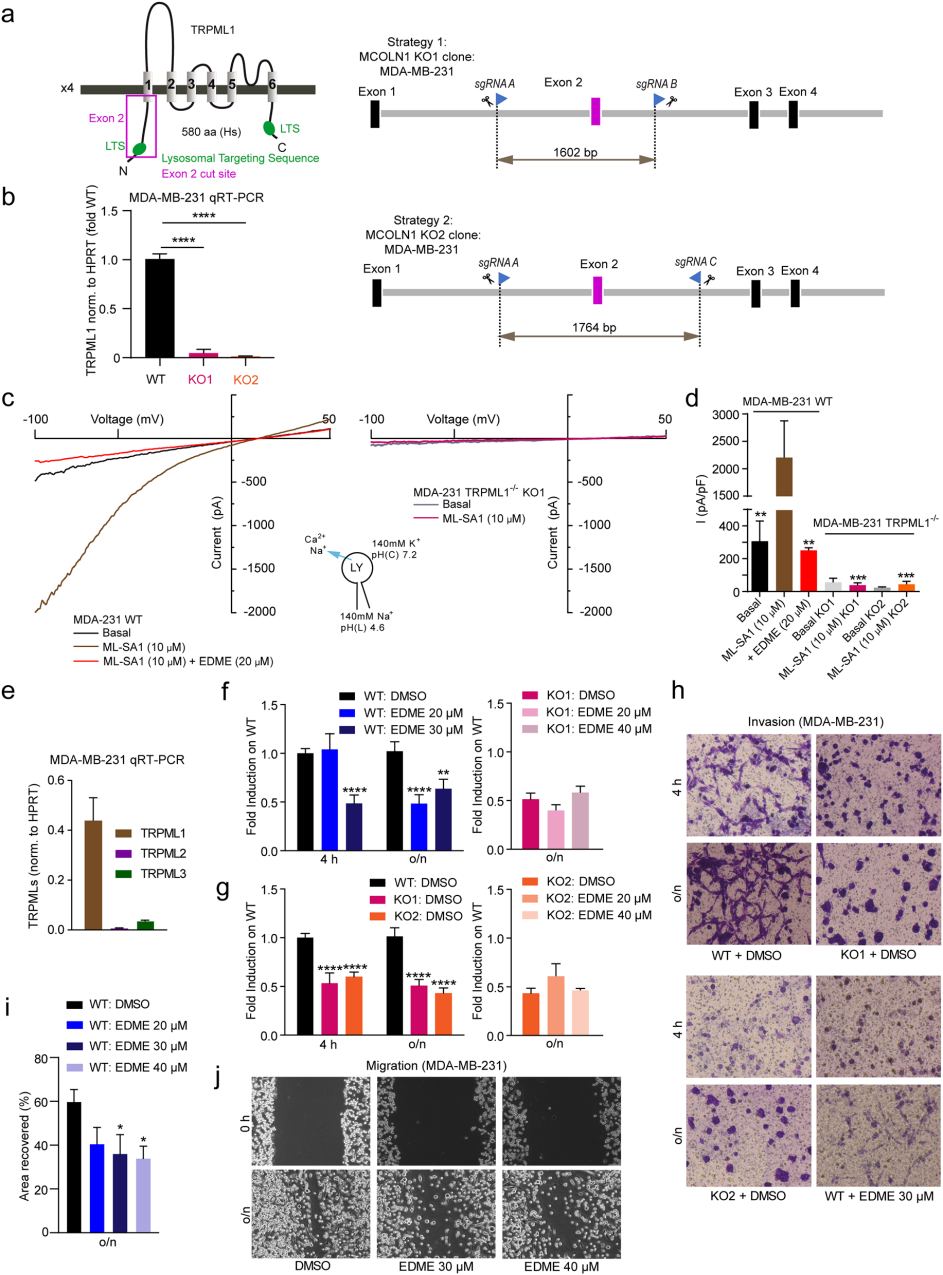
Effect of EDME on ER- breast cancer (MDA-MB-231) cell migration and invasion. (**a**) Genetic ablation of human MCOLN1 (TRPML1) in MDA-MB-231 cells was created by using two CRISPR/Cas9 strategies targeting Exon 2, resulting in KO1 and KO2. For further details see Methods section. Validation was performed by endolysosomal patch-clamp experimentation and by quantitative PCR analysis. (**b**) qRT-PCR results showing expression levels of TRPML1 in WT, KO1, and KO2 MDA-MB-231 cell lines. (**c**) Representative current densities measured from vacuolin-enlarged endo-lysosomes isolated from WT and KO1 MDA-MB-231 cells. (**d**) Statistical analysis for experiments as shown in c (n = 3, each). (**e**) qRT-PCR results showing expression levels of TRPML1, 2, and 3 in WT MDA-MB-231 cells. (**f**–**g**) Invasion assay using transwell chambers. Statistical analysis of experiments as presented in (**h**) (n = 3, each). Statistical significance was determined by two-way ANOVA followed by Bonferroni multiple comparison test. **p-value < 0.01; ***p-value < 0.0001. (**h**) Shown are representative images for WT MDA-MB-231 cells and the two TRPML1^−/−^ MDA-MB-231 cell lines KO1 and KO2 at 4 h and after o/n treatment, treated with either DMSO (control vehicle) or EDME at different concentrations. (**i**, **j**) Migration/wound healing scratch assay experiments. Shown in i is the statistical analysis of experiments as presented in (**j**) at various concentrations of EDME compared to DMSO (n = 3, each). Statistical significance was determined by one-way ANOVA followed by Bonferroni multiple comparison test. *p-value < 0.05. Shown in j are representative images for WT MDA-MB-231 cells at 0 h and after o/n incubation post scratch, treated with either the control vehicle (DMSO) or EDME at different concentrations. All statistical analysis was done using GraphPadPrism software (https://www.graphpad.com/scientific-software/prism/).

The MDA-MB-231 human breast cancer cell line is one of the most commonly used breast cancer cell lines in medical research. MDA-MB-231 is a highly aggressive, invasive and poorly differentiated triple-negative breast cancer (TNBC) cell line. It lacks estrogen and progesterone receptor expression as well as HER2 (human epidermal growth factor receptor 2) amplification. Knockdown of TRPML1 has been reported before to result in reduced migration and invasion of MDA-MB-231 cells^35^. We used EDME to show reduction in migration and invasion, and we generated TRPML1 KO cell lines using CRISPR/Cas9 to confirm involvement of TRPML1 and on-target effect of EDME. At the same time the lack of estrogen receptor was used to confirm estrogen receptor independent efficacy of EDME. Two TRPML1 KO cell lines (KO1 and KO2) were generated and loss of TRPML1 was confirmed by qRT-PCR and by endolysosomal patch-clamp experimentation (Fig. 7a–d). As shown before in overexpressing HEK293 cells and in endogenously expressing alveolar macrophages, EDME inhibits TRPML1 activation with ML-SA1. Both KO cell lines showed complete lack of activation by ML-SA1, suggesting absence of TRPML1 but also TRPML2 and TRPML3, which would likewise be activated by the non-selective TRPML-agonist ML-SA1. This was further confirmed by qRT-PCR analysis (Fig. 7e). We next assessed the effect of EDME on migration and invasion of MDA-MB-231 cells. Invasion was found to be significantly reduced after application of EDME at various concentrations (Fig. 7f–h). Effects were comparable to those seen in MDA-MB-231 TRPML1 KO cells (KO1 and KO2). Importantly, the effect was not further reduced in KO cells, indicating on-target activity of EDME (Fig. 7f,g). Like invasion migration was also reduced significantly after application of EDME at various concentrations (Fig. 7i,j). In sum these experiments confirmed estrogen receptor independent and TRPML1-mediated activity of EDME.

## Discussion

We describe here the first in-class TRPML1-selective inhibitors, which are blocking endogenously expressing TRPML1 and efficiently interfere with major functional activities of TRPML1, autophagy regulation and TFEB translocation to the nucleus. Autophagy is a vital process, involved in various diseases such as neurodegenerative, metabolic and infectious diseases as well as cancer. Both reduced and increased autophagy can impact disease. Autophagy is regulated by many factors including glucose, growth factors, amino acids, or starvation and energy status. The elimination of unwanted protein aggregates and damaged organelles is essential to avoid cell damage, e.g. damage to neurons and other brain cells, resulting eventually in a neurodegenerative processes. Pharmacological activation of autophagy processes has been propagated for the treatment of metabolic diseases such as obesity (activation of liver autophagy) or neurodegenerative diseases caused by the accumulation of inclusions or aggregates of different proteins as in the case of Parkinson’s, Huntington’s, or Alzheimer’s disease to enhance clearance and to prevent cell death. In cancer, autophagy seems to function as a tumor suppressor that prevents tumor initiation but also as a pro-survival factor helping tumor cells to endure metabolic stress and to avoid cell death triggered by chemotherapeutics^39^. Despite this diametrically opposed role autophagy might play in cancer, several inhibitors of the autophagic machinery are in preclinical development^40^. TFEB is a master regulator of autophagy by promoting expression of genes involved in autophagy, it also regulates lysosomal biogenesis, lysosomal exocytosis, lysosomal positioning and energy metabolism. Lysosomal Ca^2+^ release through TRPML1 activates calcineurin, which binds and dephosphorylates TFEB, thus promoting its nuclear translocation^10^ while phosphorylated TFEB remains inactive in the cytosol. Here, we demonstrate efficacy of the estradiol-derived TRPML1 antagonist EDME and analogs, which were rationally designed based on a profound SAR analysis of numerous tested steroidal compounds, on autophagy and TFEB translocation as well as breast cancer cell migration and invasion. To confirm that these effects were estrogen receptor independent we used the estrogen receptor negative MDA-MB-231 human breast cancer cell line. In sum, we identified novel potent and selective inhibitors for TRPML1, which effectively block autophagy and TFEB translocation as well as migration and invasion of TRPML1 endogenously expressing cells. Despite some activity of EDME at ERα, the experiments in ER negative MDA-MB-231 breast cancer cells demonstrate estrogen receptor independent effects of EDME similar to effects seen in TRPML1 KO MDA-MB-231 cells. Furthermore, we have generated the EDME analogs PRU-10 and PRU-12, which show comparable TRPML isoform selectivity but further reduced efficacy at ERα.

## Materials and methods

### Compound screening and generation of concentration response curves

To identify inhibitors of TRPML1 and TRPML3, the Spectrum Collection compound library (MS Discoveries; 2000 compounds) and additional 430 bioactive compounds were screened. To this end, fluorometric Ca^2+^ influx assays were performed by using the calcium dye Fluo-4/AM (Invitrogen, Thermo Fisher Scientific, Waltham, MA, USA) and a custom-made fluorescence imaging plate reader built into a robotic liquid handling station (Freedom Evo 150, Tecan, Männedorf, Switzerland). All measurements were done in a HEPES buffered solution (HBS), containing 132 mM NaCl, 6 mM KCl, 1 mM MgCl_2_, 1 mM CaCl_2_, 5.5 mM d-glucose, 10 mM HEPES, pH 7.4. Screening compounds dissolved in DMSO (10 mM) were prediluted in 150 mM NaCl, 20 mM HEPES, pH 7.4, to a concentration of 200 µM. For primary screens, HEK293 stably expressing plasma membrane-targeted TRPML1 or TRPML3 were trypsinized and resuspended in cell culture medium supplemented with 4 µM Fluo-4/AM. After incubation at 37 °C for 30 min, the cell suspension was briefly centrifuged resuspended in HBS, and dispensed into black pigmented, clear-bottom 384-well microwell plates (Greiner μClear, Frickenhausen, Germany). Plates were placed into the FLIPR and fluorescence signals (excitation 470 nm, emission 515 nm) were recorded with a Zyla 5.5 camera (Andor, Belfast, UK) and the μManager software like previously described^41^. In a first video, compound libraries were successively added to the cells with the Tecan 96-tip multichannel arm to a final concentration of 20 µM to map unspecific effects like autofluorescence or toxicity. In a second step the agonist ML-SA1 was pipetted in each well and fluorescence signals were recorded for 10 min. Analyses were done by calculating fluorescence intensities for each well and background areas with ImageJ (National Institutes of Health, Bethesda, MD, USA). Finally, the background was subtracted and the fluorescence intensities were normalized to initial intensities (F/F0). Concentration response curves were generated by the same procedure but compounds were manually prediluted in 96-well plates containing HBS, now supplemented with 0.1% bovine serum albumin. Here data was fitted to a four-parameter Hill equation to obtain Imin, Imax, IC_50_), and the Hill coefficient n.

### Synthesis of compounds

EDME analogs were prepared starting from commercially available 17β-estradiol (E2) and 17α-estradiol (alfatradiol) as described in detail in the Supporting Information. All compounds were fully characterized by ^1^H- and ^13^C-NMR, IR and HRMS data.

### Whole-cell patch-clamp experiments

For whole-cell patch-clamp experiments tetracycline-inducible hTRPML1L15/16A, L577/578A, hTRPML2 and TRPML3 HEK293 cell lines were used, generated by ICAGEN Inc. using the FLP-in-Tet-On system. Experiments were performed as described previously^20,42^. In brief, cells were cultured on coverslips (Fisherbrand, Fisher Scientific) in complete DMEM medium with 10% FBS (Thermo Fisher). HEK293 cells expressing the desired channel protein were patch-clamped after overnight induction with tetracycline (1 µg/mL). Patch‐clamp pipettes were pulled from glass capillaries (Sutter Instrument) using a micropipette puller (P‐87; Sutter Instrument) and had a resistance of 3–5 MΩ when filled with the pipette solution. The pipette solution contained (mM) 122 Cs‐methanosulfonate, 4 NaCl, 10 EGTA, 2 Na-ATP, 2 MgCl_2_ and 20 HEPES, pH 7.2 (with CsOH). The standard bath solution contained (mM) 153 NaCl, 5 KCl, 2 CaCl_2_, 1 MgCl_2_, 20 HEPES and 10 Glucose (pH 7.4 with NaOH). The low pH bath solution contained (mM) 150 Na-Gluconate, 5 KCl, 2 CaCl_2_, 1 MgCl_2_, 10 glucose, 10 HEPES and 10 MES (pH 4.6). Whole‐cell currents were recorded using a Multiclamp 700B amplifier (Molecular Devices) with low‐pass filtering at 3 kHz. The currents were digitized at a sampling frequency of 10 kHz using BNC-2110 and PCI-6221 with NI-DAQmx (National Instruments) and stored directly to a hard drive. Current recording was done with WinWCP5.2.7 (University of Strathclyde, UK) software, and analysis was done with the help of a customized Igor pro program (WaveMetrics). The current was recorded by 1 s rapid alterations of membrane potential (RAMP) from − 100 to + 100 mV from a holding potential of 0 mV. RAMPs were spaced at 4‐s intervals. The current recorded at − 100 mV was used for current measurement. Concentration–response curves were fitted by using the model where X is the concentration and Y is the normalized response Y = 100/(1 + 10^(X-LogIC50)).

### Endolysosomal patch-clamp experiments

For whole-LE/LY manual patch-clamp recordings, cells were treated with 1 μM vacuolin-1 (> 2 h) at 37 °C and 5% CO_2_ and experiments performed as described previously^19,25^. Compound was washed out before patch-clamp experimentation. Currents were recorded using an EPC-10 patch-clamp amplifier (HEKA, Lambrecht, Germany) and PatchMaster acquisition software (https://www.heka.com/). Data were digitized at 40 kHz and filtered at 2.8 kHz. Fast and slow capacitive transients were cancelled by the compensation circuit of the EPC-10 amplifier. All recordings were obtained at room temperature and were analyzed using PatchMaster acquisition software (https://www.heka.com/) and OriginPro 6.1 (https://www.originlab.com/). Recording glass pipettes were polished and had a resistance of 4–8 MΩ. For all experiments, salt-agar bridges were used to connect the reference Ag–AgCl wire to the bath solution to minimize voltage offsets. Liquid junction potential was corrected. For the application of the small molecule inhibitors/flavonoids, cytoplasmic solution was completely exchanged by cytoplasmic solution containing compound. The current amplitudes at − 100 mV were extracted from individual ramp current recordings. Unless otherwise stated, cytoplasmic solution contained 140 mM K-MSA, 5 mM KOH, 4 mM NaCl, 0.39 mM CaCl_2_, 1 mM EGTA and 10 mM HEPES (pH was adjusted with KOH to 7.2). Luminal solution contained 140 mM Na-MSA, 5 mM K-MSA, 2 mM Ca-MSA, 1 mM CaCl_2_, 10 mM HEPES and 10 mM MES (pH was adjusted with methanesulfonic acid to 4.6). In all experiments, 500-ms voltage ramps from -100 to + 100 mV were applied every 5 s. All statistical analysis was done using GraphPadPrism software.

### Transfection, cell culture and calcium imaging

Single cell Ca^2+^ imaging experiments were performed using Fura-2 as previously described^42^. HEK293 cells stably expressing hTRPML1∆NC-YFP, hTRPML2-YFP, hTPPML3-YFP or hTPC2L11A/L12A-RFP (22, 24) were cultured at 37 °C with 5% of CO_2_ in Dulbecco’s modified Eagle medium (Thermo Fisher), supplemented with 10% fetal bovine serum, 100 U/mL penicillin, and 0.1 mg/mL streptomycin. Cells were plated onto poly-L-lysine (sigma)-coated glass coverslips and grown for 2–3 days. For Ca^2+^ imaging experiments cells were loaded for 1 h at room temperature with Fura-2 AM (4.0 µM) and 0.005% (v/v) pluronic acid (both from Thermo Fisher) in HEPES-buffered solution (HBS) comprising 138 mM NaCl, 6 mM KCl, 2 mM MgCl_2_, 2 mM CaCl_2_, 10 mM HEPES, and 5.5 mM d-glucose (adjusted to pH 7.4 with NaOH). After loading, cells were washed with HBS and mounted in an imaging chamber. All recordings were performed in HBS. Ca^2+^ imaging was performed using a Leica DMi8 live cell microscope. Fura-2 was excited at 340 nm/380 nm. Emitted fluorescence was captured using 515 nm long-pass filter. Compounds were pre diluted in DMSO and stored as 10 mM stock solutions at − 20 °C, not exceeding three months. Working solutions were prepared directly before using by dilution with HBS.

For endolysosomal patch-clamp experiments murine alveolar macrophages were prepared and cultured as described recently^22,25^. The human TRPML2-YFP stable cell line was generated as described previously^42^.

### MDA-MB-231 cell culture and genetic ablation of human MCOLN1 (TRPML1)

MDA-MB-231 cells were grown in high glucose DMEM, supplemented with 10% FBS (Thermo Fisher), and 1% penicillin–streptomycin (Sigma-Aldrich). Cell lines were maintained at 37 °C in a 5% CO_2_ incubator. The following guide RNAs (Metabion) were designed to target Exon 2 of the human MCOLN1 gene using CRISPOR software (http://crispor.tefor.net/): sgRNA A: 5′-GGGTCCCAGCTACTAACTAC-3′; sgRNA B: 5′-GTGCAGCCATTGGGT CAACA-3′; sgRNA C: 5′-GAAAAGGGACCCAATTGTCC-3′. Approximately 3 × 105 MDA-MB-231 cells were seeded in 6-well plates (Sarstedt). The cells were co-transfected with two combinations of sgRNAs: A + B and A + C, using Lipofectamine 3000 (Themo Fisher) reagent, according to the manufacturer’s instructions. Antibiotic selection with Puromycin and Blasticidin (Gibco) was carried out for 72 h, followed by single cell dilution and clonal expansion of cells in collagen-coated 96-well plates (Sarstedt). The clones were screened and validated through several methods: first, gDNA isolation (PureLink Genomic DNA Mini Kit, Thermo Fisher), PCR (Q5 High-Fidelity DNA Polymerase, NEB) and agarose gel electrophoresis; second, isolation of RNA (Rneasy Mini Kit, Qiagen), cDNA synthesis (RevertAid First Strand cDNA synthesis kit, Thermo Fisher) and qPCR (LightCycler 480 SYBR Green I Master, Roche Life Science); third, measuring TRPML1 channel activity via the endolysosomal patch clamp technique. The strategy insured the genetic knockout of Exon 2 of human MCOLN1, its first transmembrane domain, the lysosomal targeting sequence (LTS), and a pre-mature stop codon creating a frameshift mutation.

### Estrogen receptor assay

The yeast estrogen receptor assay (YES-assay) was provided by Dr. J.P. Sumpter (Brunel University, Uxbridge, UK; Routledge & Sumpter, 1996) and was used to determine the relative transactivation activity of the human ERα in response to test substances as previously described^35^. Briefly, *Saccharomyces cerevisiae* stably transfected with a human ERα construct and an estrogen responsive element fused to the reporter gene lacZ encoding for β-galactosidase were treated with the test substances for 48 h. The β-galactosidase enzymatic activity was measured in a colorimetric assay using the substrate chlorophenol red β-d-galactopyranoside (Roche Diagnostics, Mannheim Germany). Formation of chlorophenol red was measured at 540 nm. For the test, all compounds were diluted in DMSO. 17β-estradiol (10 nM; Sigma, Deisenhofen, Germany) served as positive control and DMSO was used as vehicle control. All compounds, were dose dependently tested in a concentration range of 0.01–100 µM, using technical quadruplicates and biological triplicates.

### Autophagy assays and TFEB shift

5 × 104 wild-type human fibroblasts were seeded in a 12-well plate and treated overnight with DMSO or different TRPML1 inhibitors (EDME, PRU-10, PRU-12). The day after, cells were treated for 3 h in full media (Fed) or HBSS supplemented with 10 mM HEPES (nutrient starved) in presence of DMSO or different TRPML1 inhibitors (EDME, PRU-10, PRU-12). For autophagic flux experiments, cells were co-treated with 100 nM of bafilomycin A1 (Sigma).

Total cell lysates were prepared using TRIS HCl 10 mM pH 8.0 and 0.2% SDS supplemented with protein and phosphatase inhibitors (Sigma). Protein concentration was determined using the Bradford method. After SDS–polyacrylamide gel electrophoresis (PAGE) and immunoblotting, the protein recognized by the specific antibody was visualized by chemiluminescence methods (Luminata Crescendo Western HRP substrate, Millipore) using HRP-conjugated anti-rabbit or anti-mouse secondary antibodies (Cell Signalling). Images were acquired using Li-Cor Odyssey Fc Imaging System and densitometric quantification of unsaturated images was performed using ImageJ software (NIH). Uncropped and unprocessed western blot scans are provided as Fig. S1. The following primary antibodies were used: LC3 (Novus Cat. No. NB100-2220, 1:1000 in 5% milk), TFEB (Cell Signaling Cat. No. 4240, 1:1000 in 5% BSA) and β-actin (Santa Cruz Cat. No. Sc-47778, 1: 1000 in 5% BSA).

### Wound healing/migration and invasion assays

Wound healing assay was performed using 12-well plates (Sarstedt) at full confluency. Cells were incubated overnight (serum-free), and a scratch was performed using a yellow pipet tip. Pictures were taken at 0 h and after over night (o/n) incubation using an inverted microscope (Leica DM IL LED) and using a microscope camera (Leica DFC 3000 G). The wounded cell area was quantified using ImageJ 1.52a software and was subtracted from 0 h values.

For invasion measurements transwell chambers in 24-well permeable support plates (Corning, #3421) were coated with Corning Matrigel basement membrane matrix (Corning, #354234) for 1.5 h. A total of 4 × 104 MDA-MB-231 WT and TRPML1 KO cells were seeded on top of the chambers in serum-free medium, and direct stimulation with EDME was performed. The lower compartment contained the chemotactic gradient, medium with 10% FBS. Cells were allowed to migrate for 4 h and o/n, and were then fixed and stained with crystal violet containing methanol. Non-invaded cells were removed with Q-tips and pictures were taken of the bottom side of the membrane using an inverted microscope (Olympus CKX41) and an Olympus SC50 camera (Olympus). The number of invaded cells was quantified using ImageJ 1.52a software.

### RNA isolation and quantitative PCR

Total RNA was isolated from cells using the RNeasy Mini Kit (Qiagen). Reverse Transcription was performed using the Revert First Strand cDNA Synthesis Kit (Thermo Fisher). Real-time quantitative Reverse Transcription PCR (qPCR) was performed in triplicates for each sample using LightCycler 480 SYBR Green I Master and using the LightCycler 480 II machine (Roche Life Science), following the recommended parameters. HPRT was used as the housekeeping gene. The following human primer sets were used: HPRT; fw: 5′-TGGCGTCGTGATTAGTGATG-3′, rev: 5′-AACACCCTTTCCAAATCCTCA-3′; MCOLN1: fw: 5′-TCTTCCAGCACGGAGACAAC-3′, rev: 5′-GCCACATGAACCCCACAAAC-3′; MCOLN2: fw: 5′-AACGGTGTTTCCTGTTCCGA-3′, rev: 5′-GCCATTGCATTTCTGACG GTTA-3′; MCOLN3: fw: 5′-TGCTTCTGTGGATGGATCG-3′, rev: 5′-GAGACCATGTTC AGAGAACG-3′; TPCN1: fw: 5′-TCCCAAAGCGCTGAGATTAC-3′, rev: 5′-TCTGGTTTGAG CTCCCTTTC-3′; TPCN2: fw: 5′-GTACCCCTCTTGTGTGGACG-3′, rev: 5′-GGCCCTGACA GTGACAACTT-3′.

### TFEB immunofluorescence

3.5 × 104 HeLa cells were seeded in a 24-well plate and treated overnight with DMSO or EDME 1 µM. The day after, cells were treated for 3 h in full media (Fed) or HBSS supplemented with 10 mM HEPES (nutrient starved) in presence of DMSO or EDME 1 µM. Cells were fixed in PFA 4% 10′ and permeabilized 7´ with PBS 1X and 0.02% Triton-X. TFEB antibody (Cell Signaling Cat. No. 4240, 1:100 overnight) and Goat anti-Rabbit IgG (H + L) Secondary Antibody, Alexa Fluor 488 (ThermoFisher, 1:400 45´) were applied in blocking buffer saponin (1% BSA, 0.05% saponin and 50 mM NH4Cl in PBS1X). Samples were examined under a Zeiss LSM 880 confocal microscope. Optical sections were obtained under a 40 × immersion objective at a definition of 1024 × 1024 pixels (average of 8 scans), adjusting the pinhole diameter to 1 Airy unit for each emission channel to have all the intensity values between 1 and 254 (linear range). TFEB nuclear and cytoplasmic intensity was measured on unsaturated images using ImageJ software (NIH). The value reported is a ratio value resulting from the average intensity of nuclear TFEB fluorescence divided by the cytosolic intensity of TFEB fluorescence.

### Statistical analysis

Details of statistical analyses and n values are provided in the Materials and Methods or the Figures or Figure legends. Statistical analyses were carried out using GraphPadPrism software (https://www.graphpad.com/scientific-software/prism/). All error bars are depicted as mean ± SEM. Statistical significance is denoted on Figures as outlined in the legends.

## Supplementary Information

Supplementary Information.

## Data Availability

All data generated or analyzed during this study are included in this published article and its additional files.
