# Supplementary material for: Estradiol analogs attenuate autophagy, cell migration and invasion by direct and selective inhibition of TRPML1, independent of estrogen receptors
Source: Sci Rep. 2021 Apr 15;11:8313. doi: 10.1038/s41598-021-87817-4 (PMC8050276; doi:10.1038/s41598-021-87817-4)
Supplement: Supplementary file 1 — Supplementary Information. [file 41598_2021_87817_MOESM1_ESM.pdf]

Supplementary Information

Estradiol analogs attenuate autophagy, cell migration and invasion by direct and selective inhibition of TRPML1, independent of estrogen receptors

Philipp Rühl[1#], Anna Scotto Rosato[2#], Nicole Urban[3#], Susanne Gerndt[1], Rachel Tang[2], Carla Abrahamian[2], Charlotte Leser[1], Jiansong Sheng[4], Archana Jha[5], Günter Vollmer[6], Michael Schaefer[3*], Franz Bracher[1*], Christian Grimm[2*]

[1]*Department of Pharmacy – Center for Drug Research, Ludwig-Maximilians University, Munich, Germany*
[2]*Walther Straub Institute of Pharmacology and Toxicology, Faculty of Medicine, Ludwig-Maximilians University, Munich, Germany*
[3]*Rudolf-Boehm-Institute for Pharmacology and Toxicology, University of Leipzig, Germany*
[4]*CiPA LAB, LLC, Gaitherburg, MD, USA*
[5]*Casma Therapeutics Inc., Cambridge, MA, USA*
[6]*Institute of Zoology, Molecular Cell Physiology and Endocrinology, University of Dresden, Germany*

Supplementary Tables: 1

Supplementary Figures: 3

Supplementary Schemes: 1

Synthetic Procedures

Table S1

Potency and selectivity of PRU compounds and estradiol.

	**TRPML1**	**TRPML2**	**TRPML3**	**TRPML1:TRPML2**	**TRPML1:TRPML3**
	**IC_{50} (µM)**	**IC_{50} (µM)**	**IC_{50} (µM)**	**fold selectivity**	**fold selectivity**
**Estradiol**	5.3	30.43	>50	5.77	n.c.
**PRU-1 = EDME**	0.6	5.85	19.5	9.14	30.47
**PRU-2**	13.8	37.02	>50	2.67	n.c.
**PRU-4**	0.5	5.23	4.78	10.25	9.37
**PRU-5**	0.8	3.38	14.88	4.45	19.58
**PRU-6**	0.41	3.57	7.87	8.71	19.2
**PRU-7**	0.73	2.64	15.2	3.62	20.82
**PRU-8**	0.72	15.32	38.65	21.28	53.68
**PRU-9**	0.17	2.53	5.96	14.88	35.06
**PRU-10**	0.41	5.4	15.83	13.17	38.61
**PRU-11**	0.44	2.4	32.11	5.45	72.98
**PRU-12**	0.28	5.28	14.08	18.86	50.29

Fig. S1

Structures of the noteworthy compounds of the high-throughput screening. (a) Structures of the four specific screening hits (b) Structures of retested and pharmacologically relevant steroids.

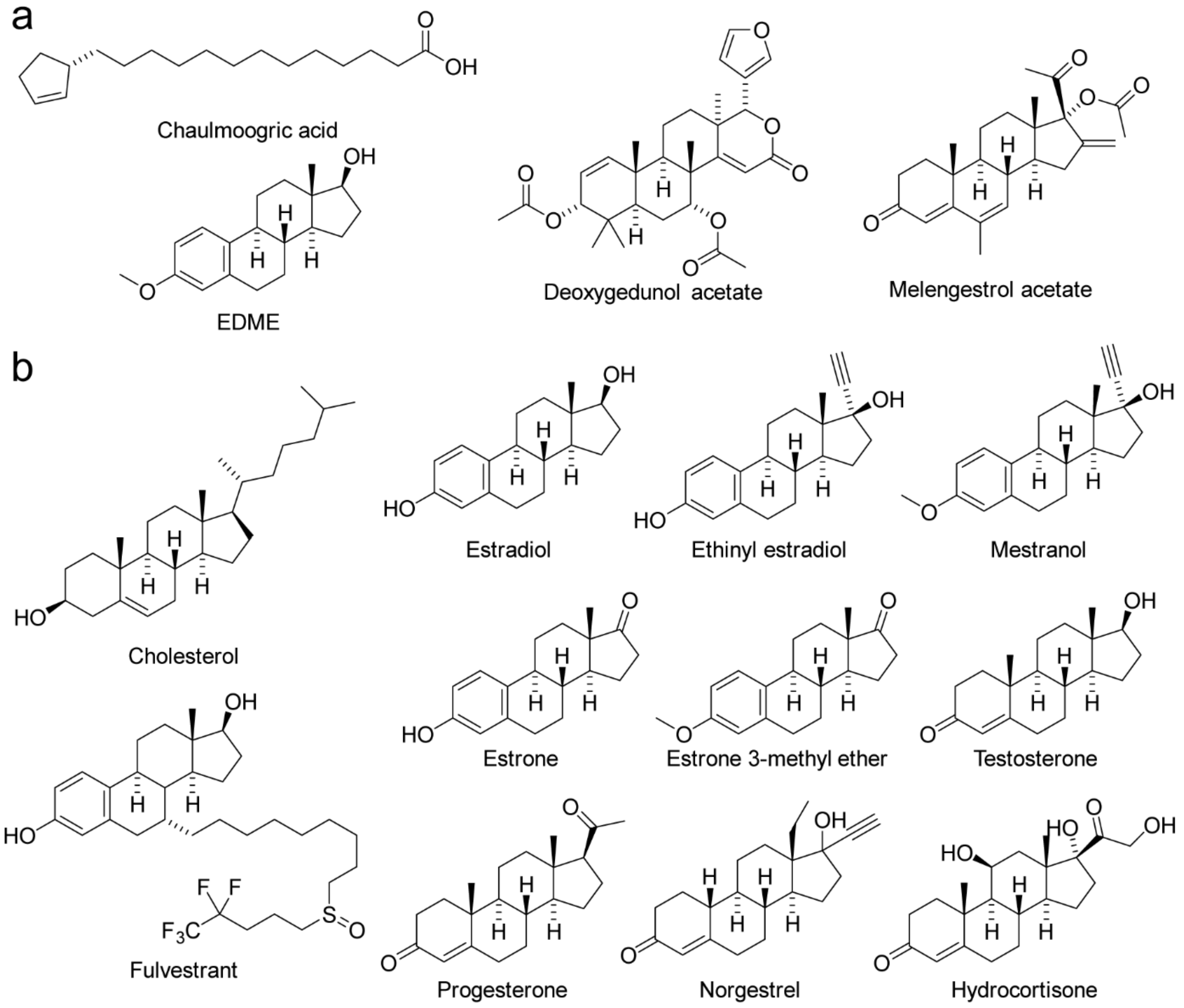

Fig. S2

Effects of PRU-10 and PRU-12 on hTPC2. Representative Fura-2 calcium signals recorded from HEK293 cells stably expressing hTPC2$^{L11A/L12A}$-RFP. Cells were either stimulated with PRU-10 or PRU-12 alone (a-b) or they were sequentially stimulated with TPC2 agonist TPC2-A1-N (10 µM) and then treated with EDME analogs (10 µM, each) (c-d). Neither an activating nor a blocking effect on TPC2 was found for PRU-10 and PRU-12.

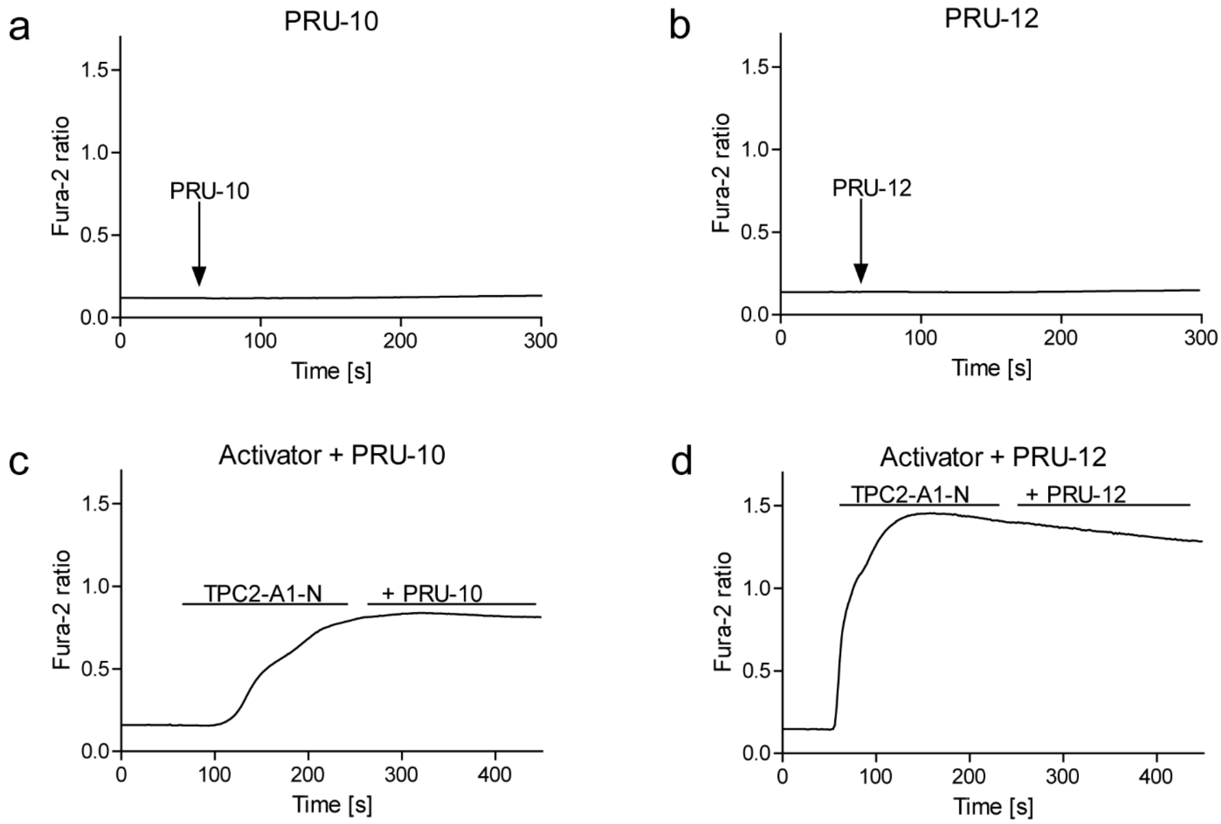

Fig. S3

Western blots uncropped. Shown are the Western blots from Fig. 6 in uncropped format.

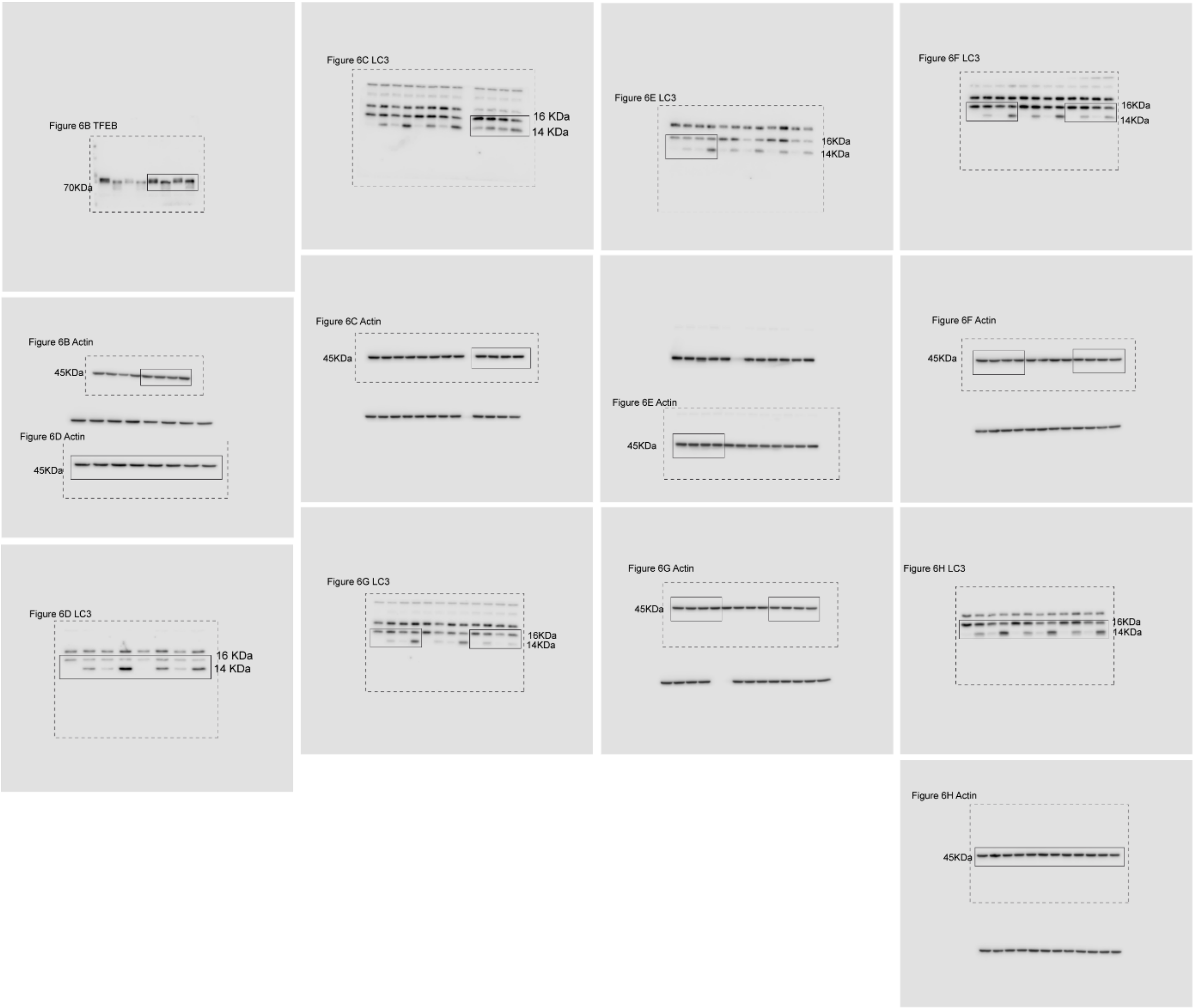

Suppl. Scheme 1

Synthesis of EDME analogs. Reaction conditions: a) for **PRU-2**: dimethyl sulfate, KOH, water/methanol, 65 °C, 1 h (51 %); b) for **PRU-4**: diethyl (bromodifluoromethyl) phosphonate, acetonitrile/water, 0 to 20 °C, 15 min (35 %); c) for **PRU-5**: bromoethane, NaOH, THF/water, reflux, 7 h (71%); d) for **PRU-6**: 2-bromopropane, NaOH, THF/water, reflux, 7 h (32 %); e) for **PRU-7**: 3-bromopropene, K_2CO_3, acetone, 50 °C, 40 h (89 %); f) for **PRU-8**: 2-(trimethylsilyl)phenyl trifluoromethanesulfonate, CsF, acetonitrile, 20 °C, 24 h (39 %); g) for **PRU-9**: 4-nitrophenyl trifluoromethanesulfonate, K_2CO_3, DMF, 20 °C, 2 h (74 %); h) for **PRU-10**: tributyl(vinyl)tin, cat. bis(triphenylphosphine)palladium(II)chloride, LiCl, 2,6-di-*tert*-butyl-4-methylphenol, DMF, N_2 atmosphere, 90 °C, 4 h (88 %); i) for **PRU-11**: phenylboronic acid, K_3PO_4, cat. $Pd(OAc)_2$, cat. SPhos, dioxane, N_2 atmosphere, 100 °C, 20 h (50 %); j) for **PRU-12**: tributyl(1-ethoxyvinyl)tin, cat. bis(triphenylphosphine) palladium(II)chloride, LiCl, DMF, N_2 atmosphere, 110 °C, 14 h, then water (54 %).

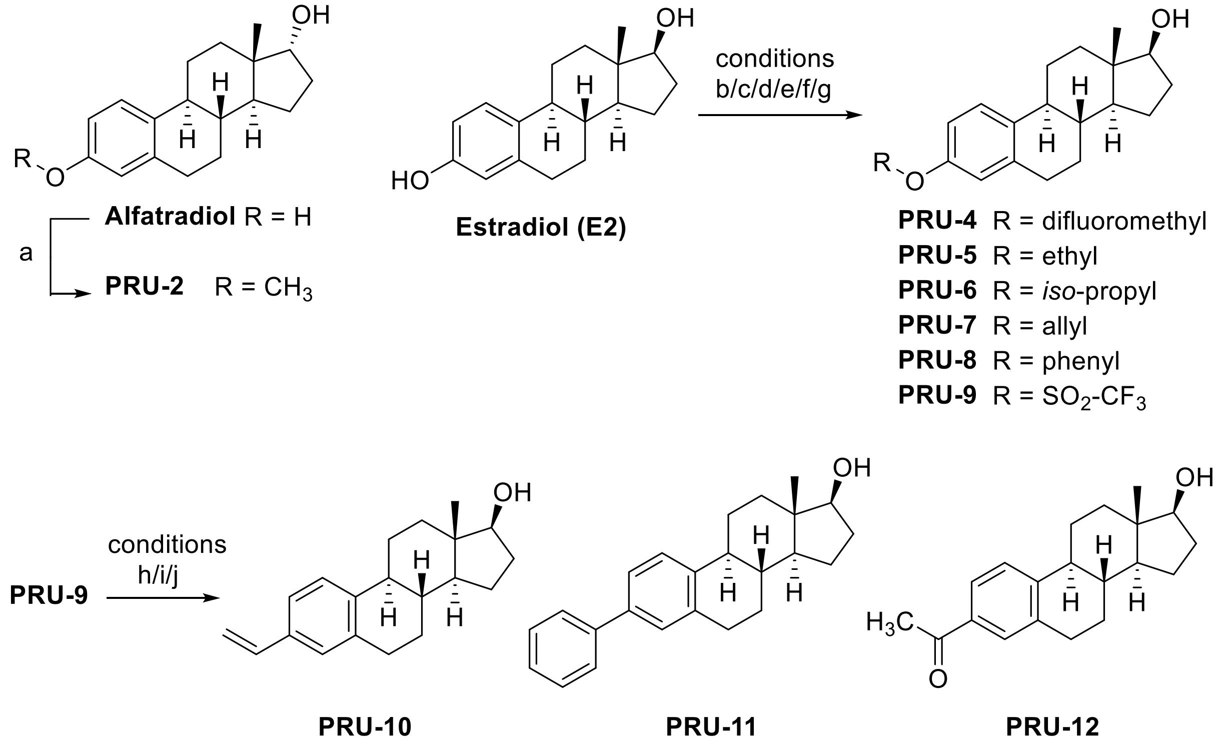

Synthetic procedures

Synthesis details and analytical data

All NMR spectra (^{1}H, ^{13}C, DEPT, H-H-COSY, HSQC, HMBC) were recorded at 23 °C on an Avance III 400 MHz Bruker BioSpin or Avance III 500 MHz Bruker BioSpin instrument unless otherwise specified. Chemical shifts *δ* are stated in parts per million (ppm) and are calibrated using residual protic solvents as an internal reference for proton ($CDCl_3$: *δ* = 7.26 ppm, DMSO: *δ* = 2.50 ppm) and for carbon the central carbon resonance of the solvent ($CDCl_3$: *δ* = 77.16 ppm, DMSO: *δ* = 39.52 ppm). Multiplicity is defined as s = singlet, d = doublet, t = triplet, q = quartet, m = multiplet. NMR spectra were analyzed with NMR software MestReNova, version 12.0.1-20560 (Mestrelab Research S.L.). High resolution mass spectra were performed by the LMU Mass Spectrometry Service applying a Thermo Finnigan MAT 95 or Joel MStation Sektorfeld instrument at a core temperature of 250 °C and 70 eV for EI or a Thermo Finnigan LTQ FT Ultra Fourier Transform Ion Cyclotron Resonance device at 250 °C for ESI. IR spectra were recorded on a Perkin Elmer FT-IR Paragon 1000 instrument as neat materials. Absorption bands were reported in wave number (cm^{-1}) with ATR PRO450-S. Melting points were determined by the open tube capillary method on a Büchi melting point B-540 apparatus and are uncorrected. HPLC purities were determined using an HP Agilent 1100 HPLC with a diode array detector and an Agilent Poroshell column (120 EC-C18; 3.0 × 100 mm; 2.7 micron) with acetonitrile/water as eluent (70:30 acetonitrile/water). All chemicals used were of analytical grade. 17β-Estradiol was purchased from TCI Deutschland GmbH (Eschborn, Germany), 17α-estradiol (alfatradiol, Ph. Eur. quality) from EDQM (Strasbourg, France). Isohexane, ethyl acetate and methylene chloride were purified by distillation. All reactions were monitored by thin-layer chromatography (TLC) using pre-coated plastic sheets POLYGRAM® SIL G/UV254 from Macherey-Nagel (Düren, Germany). Flash column chromatography was performed on Merck silica gel Si 60 (0.015 – 0.040 mm).

17β-Estradiol-3-methylether (EDME)

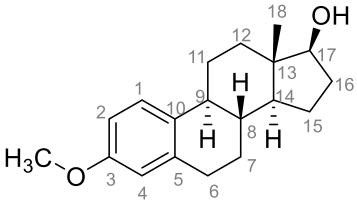

17β-Estradiol (222 mg, 0.815 mmol) was dissolved in 20 mL methanol. After addition of 5 mL of aqueous KOH (10%) and 0.50 mL (5.3 mmol) dimethyl sulfate, the solution was stirred at 65 °C for 30 min before another 0.50 mL (5.3 mmol) of dimethyl sulfate was added. After further 30 min, the solution was allowed to cool to room temperature and diluted with 20 mL of water. The precipitated solid was collected by filtration, washed with water, dried and purified by silica

gel column chromatography (isohexane/ethyl acetate 3:1) to give the methyl ether **EDME** as a colorless solid (160 mg, 0.559 mmol, 69%). m.p.: 121°C[1]: 119-120°C]. ^{1}H NMR (400 MHz, $CDCl_3$) δ/ppm = 7.21 (d, J = 8.4 Hz, 1H, 1-H), 6.71 (dd, J = 8.6 Hz, 2.8 Hz,1H, 2-H), 6.63 (d, J = 2.7 Hz, 1H, 4-H), 3.78 (s, 3H, 3-OCH_3), 3.73 (dd, J = 9.0 Hz, 8.0 Hz, 1H, 17α-H), 2.85 (m, 2H, 6-α-H, 6-β-H), 2.32 (m, 1H, 11-H), 2.19 (m, 1H, 9-H), 2.12 (m, 1H, 16-H), 1.95 (m, 1H, 12-H), 1.88 (m, 1H, 7-H), 1.70 (m, 1H, 15-H), 1.51 (m, 1H, 11-H), 1.46 (m, 1H, 16-H), 1.43 (m, 1H, 8-H), 1.39 (m, 1H, 15-H), 1.33 (m, 1H, 7-H), 1.29 (m, 1H, 12-H), 1.20 (m, 1H, 14-H), 0.71 (s, 3H, 18-H). ^{13}C NMR (100 MHz, $CDCl_3$) δ/ppm = 157.6 (C3), 138.1 (C5), 132.8 (C10), 126.5 (C1), 114.0 (C4), 111.6 (C2), 82.1 (C17), 55.4 (3-OCH_3), 50.2 (C14), 44.1 (C13), 43. (C9), 39.0 (C8), 36.9 (C12), 30.8 (C16), 30.0 (C6), 27.4 (C7), 26.5 (C11), 23.3 (C15), 11.2 (C18). IR (ATR): $\tilde{\nu}_{max}/cm^{-1}$ = 3410, 2920, 2866, 1605, 1574, 1500, 1477, 1444, 1390, 1344, 1310, 1281, 1254, 1232, 1182, 1158, 1133, 1115, 1075, 1055, 964, 947, 862, 846, 818, 777, 572, 486, 442. HRMS (EI): calcd. for $C_{19}H_{26}O_2$ $(M)^{\bullet+}$: 286.1927; found: 286.1927. Purity (HPLC): >96% (λ = 210 nm), 93% (λ = 254 nm).

17α-Estradiol-3-methylether (PRU-2)

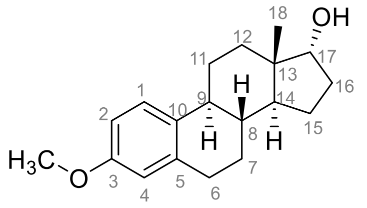

Prepared from 17α-estradiol (alfatradiol) (179 mg, 0.657 mmol) in the same manner as described above for the 17β epimer **EDME**. 17α-Estradiol-3-methylether (**PRU-2**) was obtained as a colorless solid (95.5 mg, 0.333 mmol, 51 %). m.p.: 109°C[2]: 106-107°C]. ^{1}H NMR δ/ppm = 7.18 (d, J = 8.6 Hz, 1H, 1-H), 6.67 (dd, J = 8.6 Hz, 2.8Hz,1H, 2-H), 6.59 (d, J = 2.8 Hz, 1H, 4-H), 4.34 (d, J = 4.2 Hz, 1H, OH) 3.68 (s, 3H, OCH_3), 3.57 (dd, J = 5.8 Hz, 4.1 Hz, 1H, 17β-H), 2.78 (m, 2H, 6α-H, 6β-H), 2.30 (m, 1H, 11-H), 2.10 (m, 1H, 9-H), 2.04 (m, 1H, 16-H), 1.83 (m, 1H, 7-H), 1.76 (m, 1H, 12-H), 1.71 (m, 1H, 15-H), 1.55 (m, 1H, 14-H), 1.44 (m, 1H, 12-H), 1.38 (m, 1H, 16-H), 1.34 (m, 1H, 11-H), 1.31 (m, 1H, 7-H), 1.27 (m, 1H, 8-H), 1.16 (m, 1H, 15-H), 0.61 (s, 3H, 18-H). ^{13}C NMR δ/ppm = 157.0(C3), 137.4 (C5), 132.3 (C10), 126.2 (C1), 113.4 (C4), 111.4 (C2), 78.0(C17), 54.8 (OCH_3), 47.2 (C14), 45.0(C13). 43.4 (C9), 38.8 (C8), 32.1 (C16), 31.5 (C12), 29.4 (C6), 27.8 (C7), 26.0 (C11), 23.9 (C15), 17.0 (C18). IR (ATR): $\tilde{\nu}_{max}/cm^{-1}$ = 3599, 3513, 3324, 2910, 2862, 1608, 1576, 1499, 1467, 1378, 1280, 1254, 1235, 1155, 1119, 1104, 1075, 1037, 970, 941, 683, 663, 441. HRMS (EI): calcd. for $C_{19}H_{26}O_2$ $(M)^{\bullet+}$: 286.1927; found: 286.1920. Purity (HPLC): >96% (λ = 210 nm), >96% (λ = 254 nm)

The PRU numbers arise from consecutive numbering of synthesis experiments in our electronic lab book. Experiment **PRU-3** did not lead to an identifiable product. We attempted here to prepare an EDME analogue in which the metabolically labile methoxy group is replaced by a trifluoromethoxy group. Since (based on clear evidence from literature) the same stability can as well be achieved by a difluoromethoxy analogue (= PRU-4), compound PRU-3 was no longer persued.

17β-Estradiol-3-(difluoromethyl)ether (PRU-4)

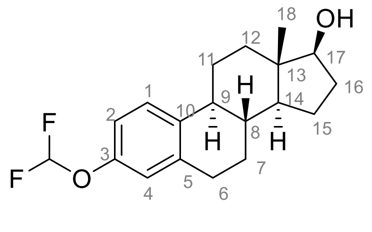

17β-Estradiol (300 mg, 1.10 mmol, 1.00 eq) and KOH (1.27 g, 22.6 mmol, 20.5 eq) were combined in a round-bottom flask following the addition of 30 mL of an acetonitrile/ water mixture (1:1). After cooling down to 0°C, diethyl (bromodifluoromethyl)phosphonate (600 mg, 2.25 mmol, 2.05 eq) was added and the biphasic mixture was allowed to reach room temperature over 15 minutes. The reaction mixture was diluted with 10 mL of diethyl ether, the layers were separated and the aqueous layer was further extracted with diethyl ether (3 × 10 mL). The combined organic layers were dried over anhydrous Na_2SO_4 and concentrated *in vacuo*. Purification was accomplished by silica gel column chromatography (isohexane/ethyl acetate 3:1) to yield **PRU-4** as a colorless oil (125 mg, 0.388 mmol, 35%). Analytical data are in accordance with literature[3]. ^{1}H NMR *δ*/ppm = 7.30 (d, *J* = 8.50 Hz, 1H, 1-H), 7.14 (t, *J* = 74.50 Hz,1H, CHF_2), 6.90 (dd, *J* = 8.5, 2.7 Hz, 1H, 2-H), 6.85 (d, *J* = 2.7 Hz,1H, 4-H), 4.50 (d, *J* = 4.8 Hz,1H, OH), 3.52 (m, 1H, 17-α-H), 2.80 (m, 2H, 6-α-H, 6-β-H), 2.28 (m, 1H, 11-H), 2.13 (m, 1H, 9-H), 1.88 (m, 1H, 16-H), 1.83 (m, 1H, 12-H), 1.79 (m, 1H, 7-H), 1.59 (m, 1H, 15-H), 1.38 (m, 1H, 16-H), 1.35 (m, 1H, 11-H), 1.32 (m, 1H, 8-H), 1.29 (m, 1H, 7-H), 1.26 (m, 1H, 15-H), 1.20 (m, 1H, 12-H), 1.14 (m, 1H, 14-H), 0.66 (s, 3H, 18-H). ^{13}C NMR *δ*/ppm= 148.7 (C3), 138.4 (C5), 137.2 (C10), 126.8 (C1), 118.7 (C4), 116.5 (t, *J* = 257 Hz, CHF_2), 116.0 (C2), 80.0 (C17), 49.5 (C14), 43.6 (C9), 42.8 (C13), 38.2 (C8), 36.5 (C12), 29.9 (C16), 29.0(C6), 26.6 (C7), 25.9 (C11), 22.8 (C15), 11.2 (C18). IR (ATR): $\tilde{\nu}_{max}/cm^{-1}$ =3410, 2929, 2870, 1611, 1496, 1452, 1382, 1356, 1233, 1165, 1128, 1047, 934, 878, 821, 792, 763, 575, 449. HRMS (EI): calcd. for $C_{19}H_{24}F_2O_2$ $(M)^{•+}$: 322.1739; found: 322.1749. Purity (HLPC): >96% (λ = 210 nm), >96% (λ = 254 nm).

17β-Estradiol-3-ethylether (PRU-5)

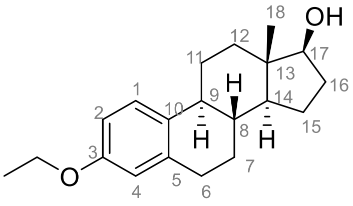

Bromoethane (118 mg, 1.08 mmol, 1.35 eq) was added to a solution of 17β-estradiol (217 mg, 0.797 mmol, 1.00 eq) and NaOH (44 mg, 1.1 mmol, 1.4 eq) in THF (20 mL) and water (5 mL). The reaction mixture was heated under reflux for 7 h and then diluted with water (10 mL). The mixture was extracted with diethyl ether (3 × 10 mL), the combined organic layers were dried over anhydrous Na_2SO_4 and concentrated *in vacuo*. The crude product was purified by column chromatography (isohexane/ethyl acetate 3:1) to yield **PRU-5** as a colorless solid (171 mg, 0.571 mmol, 71%). m.p.: 120°C[4]: 124-125°C]. ^{1}H NMR δ/ppm = 7.14 (d, J = 8.2 Hz, 1H, 1-H), 6.64 (dd, J = 8.6 Hz, 2.8 Hz, 1H, 2-H), 6.57 (d, J = 2.8 Hz, 1H, 4-H), 4.48 (d, J = 4.8, 1H, OH), 3.94 (q, J = 6.9 Hz, 2H, OCH_2), 3.51 (td, J = 8.5 Hz, 4.8 Hz 1H, 17-α-H), 2.75 (m, 2H, 6-α-H, 6-β-H), 2.25 (m, 1H, 11-H), 2.10 (m, 1H, 9-H), 1.86 (m, 1H, 16-H), 1.82 (m, 1H, 12-H), 1.78 (m, 1H, 7-H), 1.58 (m, 1H, 15-H), 1.37 (m, 1H, 16-H), 1.32 (m, 1H, 11-H), 1.29 (t, J = 7.0 Hz, 3H, ethyl CH_3), 1.27 (m, 1H, 8-H), 1.24 (m, 1H, 7-H), 1.21 (m, 1H, 15-H), 1.16 (m, 1H, 12-H), 1.10 (m, 1H, 14-H), 0.66 (s, 3H, 18-H). ^{13}C NMR δ/ppm = 156.3 (C3), 137.4 (C5), 132.1 (C10), 126.2 (C1), 114.1 (C4), 112.0(C2), 80.1 (C17), 62.7 (OCH_2), 49.6 (C14), 43.6 (C9), 42.9 (C13), 38.6 (C8), 36.6 (C12), 29.9 (C16), 29.3 (C6), 26.9 (C7), 26.1 (C11), 22.8 (C15), 14.8 (ethyl CH_3), 11.3 (C18). IR (ATR): $\tilde{\nu}_{max}$/cm^{-1} = 3410, 2920, 2866, 1605, 1500, 1477, 1390, 1344, 1310, 1254, 1232, 1182, 1157, 1133, 1054, 947, 862, 818, 777, 572. HRMS (EI): calcd. for $C_{20}H_{28}O_2$ $(M)^{\bullet+}$: 300.2089; found: 300.2082. Purity (HPLC): >96% (λ = 210 nm), >96% (λ = 254 nm).

17β-Estradiol-3-isopropylether (PRU-6)

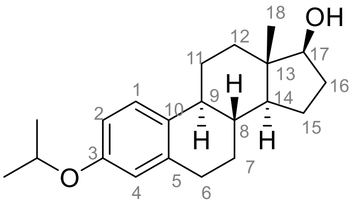

2-Bromopropane (128 mg, 1.03 mmol, 1.40 eq) was added to a solution of 17β-estradiol (200 mg, 0.734 mmol, 1.00 eq) and NaOH (41 mg, 1.10 mmol, 1.50 eq) in THF (20 mL) and water (5 mL). The solution was heated under reflux for 7 h and then diluted with water. The reaction mixture was extracted with diethyl ether (3 × 10 mL) and the combined organic layers

were dried over Na_2SO_4, filtered and concentrated *in vacuo*. Purification was accomplished by column chromatography (isohexane/ethyl acetate 3:1) to yield **PRU-6** as a colorless solid (74.9 mg 0.238 mmol, 32%). m.p.: 124°C[5]:119-122°C]. ^{1}H NMR δ/ppm = 7.13 (d, J = 8.5 Hz,1H, 1-H)), 6.63 (dd, J = 8.5 Hz, 2.7 ,1H, 2-H)), 6.56 (d, J = 2.7 Hz, 1H, 4-H), 4.50 (sept, J = 6.1 Hz, 1H, isopropyl CH), 4.48 (d, J = 4.8 Hz, 1H, OH), 3.51 (td, J = 8.5 Hz, 4.8 Hz,1H, 17-H), 2.74 (m, 2H, 6-α-H, 6-β-H), 2.24 (m, 1H, 11-H), 2.07 (m, 1H, 9-H), 1.87 (m, 1H, 16-H), 1.83 (m, 1H, 12-H), 1.77 (m, 1H, 7-H), 1.57 (m, 1H, 15-H), 1.38 (m, 1H, 16-H), 1.32 (m, 1H, 11-H), 1.29 (m, 1H, 8-H), 1.26 (m, 1H, 15-H), 1.23 (m, 1H, C7), 1.21 (2 d, J = 6.0 Hz, 2 x 3H, 2 isopropyl CH_3), 1.17 (m, 1H, 12-H), 1.11 (m, 1H, 14-H), 0.66 (s, 3H, 18-H). ^{13}C NMR δ/ppm = 155.1 (C3), 137.4 (C5), 132.0 (C10), 126.1 (C1), 115.4 (C4), 113.1 (C2), 80.0 (C17), 68.8 (isopropyl CH), 49.5 (C14), 43.5 (C9), 42.8 (C13), 38.6 (C8), 36.6 (C12), 29.9 (C16), 29.2 (C6), 26.9 (C7), 26.0 (C11), 22.8 (C15), 21.9 and 21.9 (2 isopropyl CH_3), 11.3 (C18). IR (ATR): $\tilde{\nu}_{max}$/cm^{-1} = 3478, 2914, 2863, 1610, 1494, 1378, 1333, 1279, 1252, 1109, 1052, 1008, 970, 874, 808, 774, 572. HRMS (EI): calcd. for $C_{21}H_{30}O_2$ $(M)^{\bullet+}$: 314.2240; found: 314.2241. Purity (HPLC): >96% (λ = 210 nm), >96% (λ = 254 nm).

17β-Estradiol-3-allylether (PRU-7)

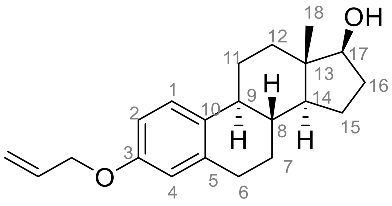

17β-Estradiol (136 mg, 0.499 mmol, 1.00 eq), 3-bromopropene (121 mg, 1.00 mmol, 86.0 µL, 2.00 eq), K_2CO_3 (248 mg, 1.50 mmol, 3.00 eq) and acetone (7.5 mL) were combined in a round bottom flask and stirred at 50 °C for 40 h. Then the mixture was diluted with water (5 mL) and extracted with CH_2Cl_2 (3 × 10 mL). The combined organic layers were dried over anhydrous Na_2SO_4 and concentrated *in vacuo*. The crude product was purified by silica gel column chromatography (isohexane/ethyl acetate 3:1) to afford **PRU-7** as a colorless solid (138 mg, 0.443 mmol, 89%). Analytical data are in accordance with literature[6]. m.p.: 64°C. ^{1}H NMR δ/ppm = 7.15 (d, J = 8.9 Hz ,1H, 1-H), 6.68 (dd, J = 8.6 Hz, 2.8 Hz,1H, 2-H), 6.61 (d, J = 2.7 Hz, 1H, 4-H), 6.01 (ddt, J = 17.4 Hz, 10.5 Hz, 5.2 Hz,1H, allyl CH), 5.36 (dq, J = 17.2 Hz, 1.8 Hz, 1H, allyl =CH_2), 5.22 (dq, J = 10.5 Hz, 1.6 Hz, 1H, 21-H), 4.49 (m, 3H, OH, OCH_2), 3.52 (td, J = 8.5 Hz, 4.9 Hz, 1H, 17-H), 2.75 (m, 2H, 6-H), 2.25 (m, 1H, 11-H), 2.09 (m, 1H, 9-H), 1.88 (m, 1H, 16-H), 1.84 (m, 1H, 12-H), 1.79 (m, 1H, 7-H), 1.58 (m, 1H, 15-H), 1.38 (m, 1H, 16-H), 1.32 (m, 1H, 11-H), 1.29 (m, 1H, 8-H), 1.26 (m, 1H, 15-H), 1.23 (m, 1H, 7-H), 1.18 (m, 1H, 12-H), 1.12 (m, 1H, 14-H), 0.66 (s, 3H, 18-H). ^{13}C NMR δ/ppm = 155.9 (C3), 137.4 (C5), 134.0 (allyl CH), 132.3 (C10), 126.1 (C1), 117.0 (allyl =CH_2), 114.3 (C4), 112.1 (C2), 80.0

(C17), 68.0(OCH$_2$), 49.5 (C14), 43.5 (C9), 42.8 (C13), 38.5 (C8), 36.6 (C12), 29.9 (C16), 29.2 (C6), 26.8 (C7), 26.0(C11), 22.8 (C15), 11.2 (C18). IR (ATR): $\tilde{\nu}_{max}$/cm^{-1} = 3399, 2923, 2359, 1606, 1498, 1455, 1381, 1345, 1309, 1282, 1230, 1158, 1133, 1053. 1023, 917, 861, 818, 788, 569. HRMS (EI): calcd. for $C_{21}H_{28}O_2$ (M)$^{•+}$: 312.2084; found: 312.2083. Purity (HPLC): >96% (λ = 210 nm), >96% (λ = 254 nm).

17β-Estradiol-3-phenylether (PRU-8)

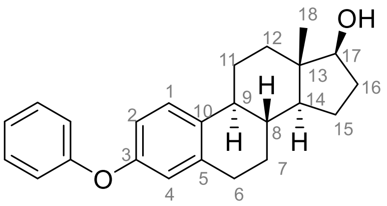

17β-Estradiol (202 mg, 0.741 mmol, 1.00 eq) and CsF (337 mg, 2.22 mmol, 3.00 eq) were suspended in acetonitrile (6.2 mL), then 2-(trimethylsilyl)phenyl trifluoromethanesulfonate (270 µL, 1.11 mmol, 1.50 eq) was added and the suspension was stirred for 24 h at room temperature. The resulting mixture was washed with brine (20 mL) and extracted with diethyl ether (3 × 10 mL). The combined ether fractions were dried over Na_2SO_4 and concentrated under reduced pressure. Purification was accomplished by column chromatography (isohexane/ethyl acetate 3:1) to yield **PRU-8** as a colorless oil (100 mg, 0.29 mmol, 39%). ^{1}H NMR *δ*/ppm = 7.36 (m, 2H), 7.27 (d, *J* = 8.5 Hz, 1H), 7.1 (m, 1H), 6.96 (m, 2H), 6.75 (dd, *J* = 8.5 Hz, 2.6 Hz, 1H, 2-H), 6.69 (d, *J* = 2.7 Hz, 1H, 4-H), 4.50 (d, *J* = 4.9Hz, 1H, OH), 3.53 (td, *J* = 8.5 Hz, 4.8 Hz, 17-H), 2.76 (m, 2H, 6-H), 2.28 (m, 1 H, 11-H), 2.15 (m, 1H, 9-H). 1.88 (m, 1H, 16-H), 1.85 (m, 1H, 12-H), 1.79 (m, 1H, 7-H), 1.59 (m, 1H, 15-H), 1.38 (m, 1H, 11-H), 1.35 (m, 1H, 16-H), 1.33 (m, 1H, 8-H), 1.29 (m, 1H, 7-H), 1.25 (m, 1H, 15-H), 1.19 (m, 1H, 12-H), 1.12 (m, 1H, 14-H), 0.68 (s, 3H, 18-H). ^{13}C NMR *δ*/ppm = 157.1, 154.1, 138.3, 135.4, 129.9, 126.8, 123.0, 118.6, 118.7, 116.1 (C2), 80.0 (C17), 49.6 (C14), 43.6 (C9), 42.8 (C13), 38.4 (C8), 36.6 (C12), 29.9 (C16), 29.1 (C6), 26.7 (C7), 25.9 (C11), 22.77 (C15), 11.2 (C18). HRMS (EI): calcd. for $C_{24}H_{28}O_2$ (M)$^{•+}$: 348.2084, found: 348.2089. Purity (HPLC): >91% (λ = 210 nm), >91% (λ = 254 nm).

17β-Estradiol-3-trifluoromethanesulfonate (PRU-9)

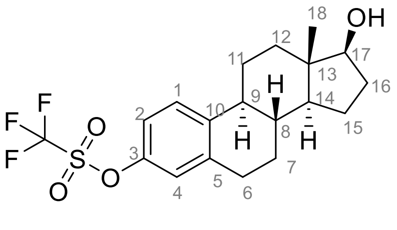

17β-Estradiol (1.00 g, 3.68 mmol, 1.00 eq) was dissolved in DMF (12 mL). K_2CO_3 (1.20 g, 7.34 mmol, 2.00 eq) was added, followed by 4-nitrophenyl trifluoromethanesulfonate (1.04 g, 3.85 mmol, 1.05 eq), and the resulting suspension was stirred for 2 h at room temperature. Then water (12 mL) and diethyl ether (5 mL) were added, the aqueous layer was separated and extracted with diethyl ether (3 × 5 mL). The combined organic layers were washed with cold 1M hydrochloric acid (10 mL), 1M NaOH solution (3 × 10 mL), water (3 × 10 mL), brine (10 mL) and 1M aqueous LiCl solution and dried over anhydrous Na_2SO_4. After evaporation the crude product was purified by column chromatography (isohexane/ethyl acetate 2:1) to yield **PRU-9** as a colorless solid (1.10 g, 2.72 mmol, 74%). m.p.: 79°C[7]: 123-125°C]. ^{1}H NMR *δ*/ppm = 7.34 (d, *J* = 8.7 Hz, 1H, 1-H), 7.02 (dd, *J* = 8.7 Hz, 2.7 Hz, 1H, 2-H), 6.97 (d, *J* = 2.7 Hz, 1H, 4-H), 3.74 (t, *J* = 8.7 Hz, 1H, 17-H), 2.89 (m, 2H, 6-H), 2.32 (m, 1H, 11-H), 2.24 (m, 1H, 9-H), 2.13 (m, 1H, 16-H), 1.98 (m, 1H, 12-H), 1.92 (m, 1H, 7-H), 1.71 (m, 1H, 15-H), 1.55 (m, 1H, 11-H), 1.49 (m, 1H, 16-H), 1.44 (m, 1H, 8-H), 1.38 (m, 1H, 15-H), 1.34 (m, 7-H), 1.30 (m, 1H, 12-H), 1.20 (m, 1H, 14-H), ^{13}C NMR *δ*/ppm = 147.6 (C3), 141.0 (C5), 139.7 (C10), 127.3 (C1), 121.3 (C4), 119.3 (q, *J* = 322.6 Hz, CF_3), 118.3 (C2), 81.9 (C17), 50.2 (C14), 44.2 (C9), 43.3 (C13), 38.4 (C8), 36.7 (C12), 30.7 (C16), 29.7 (C6), 26.9 (C7), 26.2 (C11), 23.3 (C15), 11.2 (C18). IR (ATR): $\tilde{\nu}_{max}$/cm^{-1} = 3400, 2958, 1489, 1418, 1250, 1206, 1142, 1049, 1002, 928, 976, 856, 718, 618, 499. HRMS (EI): calcd. for $C_{19}H_{23}F_3O_4S$ $(M)^{\bullet+}$: 404.1264, found: 404.1269. Purity (HPLC) >96% (λ = 210 nm), >96% (λ = 254 nm).

(8*R*,9*S*,13*S*,14*S*,17*S*)-13-Methyl-3-ethenyl-7,8,9,11,12,13,14,15,16,17-decahydro-6*H*-cyclopenta[a]phenanthren-17-ol (PRU-10)

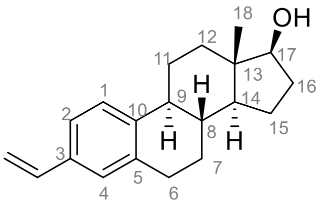

A flame-dried Schlenk flask was evacuated and backfilled with nitrogen 5 times prior to the addition of 300 mg (0.742 mmol, 1.00 eq) of 17β-estradiol-3-trifluoromethanesulfonate (**PRU-9**), 135 mg (3.15 mmol, 4.26 eq) lithium chloride, 1.6 mg of 2,6-di-*tert*-butyl-4-methylphenol and 52 mg (0.074 mmol, 0.10 eq) of bis(triphenylphosphine)palladium(II)chloride. After addition of 4.5 mL DMF, 0.28 mL (305 mg, 0.962 mmol, 1.30 eq) tributyl(vinyl)tin were added dropwise. The resulting suspension was stirred for 4 h at 90 °C under nitrogen atmosphere, then cooled to room temperature and treated with 0.3 mL of pyridine and 0.6 mL of hydrogen fluoride pyridine (Olah's reagent). The resulting mixture was stirred at room temperature overnight and then diluted with 20 mL diethyl ether, filtered and washed with 10 mL of water, 10 mL of 10% hydrochloric acid, again 10 mL of water and 10 mL of brine. The organic layer was dried over anhydrous Na_2SO_4 and concentrated under reduced pressure. The crude

product was suspended in cold isohexane and left in the fridge overnight. The precipitated solid was collected by filtration and purified by silica gel column chromatography (isohexane/ethyl acetate 4:1) to yield **PRU-10** as a colorless solid (184 mg, 0.651 mmol, 88%). Analytical data are in accordance with literature[8]. m.p.: 104°C. ^{1}H NMR *δ*/ppm = 7.24 (d, *J* = 8.2 Hz, 1H, 1-H), 7.2 (dd, *J* = 8.1 Hz, 1.8 Hz, 1H, 2-H), 7.12 (d, *J* = 1.8 Hz, 1H, 4-H), 6.64 (dd, *J* = 17.6 Hz, 10.9 Hz, 1H, vinyl CH), 5.73 (dd, *J* = 17.7 Hz, 1.2 Hz, 1H, vinyl CH_2), 5.17 (dd, *J* = 17.7 Hz, 1.2 Hz, 1H, vinyl CH_2), 4.50 (d, *J* = 4.8 Hz, 1H, 17-OH), 3.53 (td, *J* = 8.5 Hz, 4.8 Hz, 1H, 17α-H), 2.79 (dd, *J* = 8.8 Hz, 4.1 Hz, 2H, 6α-H, 6β-H), 2.29 (m, 1H, 11-H) 2.16 (m, 1H, 9-H) 1.89 (m, 1H, 16-H) 1.85 (m, 1H, 12-H) 1.81 (m, 1H, 7-H) 1.59 (m,1H, 11-H) 1.38 (m, 1H, 16-H), 1.35 (m, 1H, 11-H), 1.33 (m, 1H, 9-H), 1.28 (m, 1H, 7-H) 1.26 (m, 1H, 15-H), 1.19 (m, 1H, 12-H), 1.14 (m, 1H, 14-H), 0.67 (s, 3H, 18-H). ^{13}C NMR *δ*/ppm = 140.0 (C10), 136.6 (C19), 136.4 (C5), 134.3 (C3), 126.5 (C4), 125.4 (C1), 123.3 (C2), 113.1 (C20), 80.0 (C17), 49.6 (C8), 44.0(C14), 42.8 (C13), 38.5 (C9), 36.6 (C12), 29.9 (C16), 28.9 (C6), 26.8 (C7), 25.8 (C11), 22,8 (C15), 11.2 (C18). IR (ATR): $\tilde{\nu}_{max}/cm^{-1}$ = 3410, 2930, 2864, 1629, 1562, 1496, 1445, 1385, 1335, 1250, 1134, 1074, 1052, 1021, 988, 898, 823, 787, 727, 567, 443. HRMS (EI): calcd. for $C_{20}H_{26}O$ $(M)^{\bullet+}$: 282.1978, found: 282.1984. Purity (HPLC): >96% (λ = 210 nm), >96% (λ = 254 nm).

(8*R*,9*S*,13*S*,14*S*,17*S*)-13-Methyl-3-phenyl-7,8,9,11,12,13,14,15,16,17-decahydro-6*H*-cyclopenta[a]phenanthren-17-ol (PRU-11)

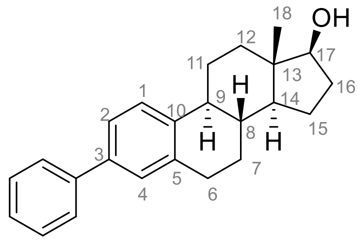

A flame dried Schlenk flask was evacuated and backfilled with nitrogen 5 times prior to the addition of 17β-estradiol-3-trifluoromethanesulfonate (**PRU-9**), 209 mg, 0.50 mmol, 1.00 eq), phenylboronic acid (122 mg, 1.00 mmol, 2.00 eq), K_3PO_4 (212 mg, 1.00 mmol, 2.00 eq), $Pd(OAc)_2$ (5.7 mg, 0.025 mmol, 0.05 eq), SPhos (20.5 mg, 0.0500 mmol, 0.100 eq) and dioxane (8 mL). The resulting suspension was heated at 100 °C for 20 h under nitrogen atmosphere and then quenched with water (15 mL) and diluted with ethyl acetate (15 mL). The organic phase was separated and the aqueous phase was extracted with ethyl acetate (3 × 10 mL). The combined organic phases were filtered, washed with brine and dried over anhydrous Na_2SO_4. The crude product was purified by column chromatography (hexane/ethyl acetate 4:1) to yield **PRU-11** as a colorless solid (174 mg, 0.501 mmol, 50%). m.p.: 172°C.^{1}H NMR *δ*/ppm = 7.58 (m, 2H), 7.42 (m, 2H), 7,39 (m, 2H, 1-H, 2-H), 7.33 (m, 2H), 3.76 (dd, *J* = 9.0 Hz, 7.9 Hz ,1H, 17α-H), 2.96 (m, 2H, 6-H), 2.40 (m, 1H, 11-H), 2.30 (m,1H, 9-H), 2.15 (m, 1H, 16-H),

1.99 (m,1H, 12-H), 1.94 (m, 1H, 7-H), 1.74 (m, 1H, 15-H), 1.58 (m, 1H, 11-H), 1.52 (m, 1H, 16-H), 1,50 (m, 1H, 8-H), 1,43 (m, 1H, 7-H), 1.40 (m, 1H, 15-H), 1.35 (m, 1H, 12-H), 1.27 (m, 1H, 14-H), 0.81 (s, 3H, 18-H). ^{13}C NMR *δ*/ppm = 141.3, 139.7 (C10), 138.7 (C3), 137.3 (C5), 128.8, 127.9 (C4), 127.2, 127.1, 126.0(C1), 124.6 (C2), 82.1 (C17), 50.3 (C14), 44.5 (C9), 43.4 (C13), 38.8 (C8), 36.9 (C12), 30.8 (C16), 29.8 (C6), 27.4 (C7), 26.3 (C11), 23.3 (C15), 11.2 (C18). IR (ATR): $\tilde{\nu}_{max}$/cm^{-1} = 3563, 2935, 2898, 1484, 1376, 1127, 1067, 1028, 1008, 889, 849, 762, 713, 698, 529. HRMS (EI): calcd. for $C_{24}H_{28}O$ $(M)^{\bullet+}$: 332.2135, found: 332.2132. Purity (HPLC): >95% (λ = 210 nm), >95% (λ = 254 nm)

1-((8*R*,9*S*,13*S*,14*S*,17*S*)-17-Hydroxy-13-methyl-7,8,9,11,12,13,14,15,16,17-decahydro-6*H*-cyclopenta[a]phenanthren-3-yl)ethan-1-one (PRU-12)

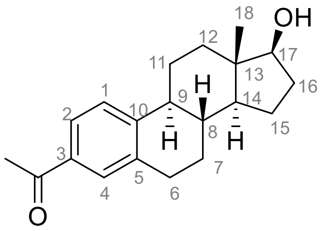

A flame dried Schlenk flask was evacuated and backfilled with nitrogen 5 times prior to the addition of 202 mg (0.50 mmol, 1.00 eq) 17β-estradiol-3-trifluoromethane-sulfonate (PRU-9), 64.2 mg (1.50 mmol, 3.00 eq) lithium chloride and 35.1 mg (0.0500 mmol, 0.100 eq) bis(triphenylphosphine)palladium(II)chloride. After addition of 3 mL of DMF, tributyl(1-ethoxyvinyl)tin (0.170 mL, 181 mg, 0.503 mmol, 1.00 eq) was added dropwise and the resulting suspension was stirred at 110 °C for 14 h under nitrogen atmosphere. After cooling, the mixture was treated with 5 mL of water and diluted with 5 mL of ethyl acetate. The organic phase was separated and washed with cold water (3 × 10 mL), brine and aqueous 1M LiCl solution, dried over anhydrous Na_2SO_4 and concentrated *in vacuo*. The crude product was purified by flash column chromatography (isohexane/ ethyl acetate 4:1) to yield PRU-12 as a colorless solid (80.2 mg, 0.27 mmol, 54%). m.p.: 180°C[8]:170-172°C]. ^{1}H NMR *δ*/ppm = 7.72 (dd, *J* = 8.1 Hz, 2.0 Hz, 1H, 2-H), 7.68 (d, *J* = 1.9 Hz, 1H, 4-H), 7.38 (d, *J* = 8.3Hz, 1H, 1-H), 3.74 (dd, 9.0 Hz, 8.0 Hz, 17α-H), 2.92 (m, 2H, 6α-H, 6β-H), 2.57 (s, 3H, acetyl CH_3), 2.37 (m, 1H, 11-H), 2.28 (m, 1H, 9-H), 2.13 (m, 1H, 16-H), 1.98 (m, 1H, 12-H), 1.93 (m, 1H, 7-H), 1.72 (m, 1H, 15-H), 1.55 (m, 1H, 11-H), 1.50 (m, 1H, 16-H), 1.46 (m, 1H, 8-H), 1.39 (m, 1H, 15-H), 1.35 (m, 1H, 7-H), 1.30 (m, 1H, 12-H), 1.22 (m, 1H, 14-H), 0.79 (s, 3H, 18-H). ^{13}C NMR *δ*/ppm = 198.4 (C=O), 146.3 (C10), 137.3 (C5), 134.8

(C3), 129.1 (C4), 125.8 (C2), 125.7 (C1), 81.9 (C17), 50.3 (C14), 44.9 (C9), 43.3 (C13), 38.5 (C8), 36.8 (C12), 30.7 (C16), 29.6 (C6), 27.1 (C7), 26.7 (acetyl CH_3), 26.1 (C11), 23.3 (C15), 11.2 (C18). IR (ATR): $\tilde{\nu}_{max}/cm^{-1}$ = 3507, 2917, 2868, 1664, 1605, 1562, 1363, 1267, 1171, 1056, 898, 834, 592. HRMS (EI): calcd. for $C_{20}H_{26}O$ $(M)^{\bullet+}$: 298.1927, found: 298.1926. Purity (HPLC): >96% (λ = 210 nm), >96% (λ = 254 nm).

Supporting Information References:

1. Muddana, S.S., Price, A.M., MacBride, M.M., and Peterson, B.R. (2004). 11beta-alkyl-Delta9-19-nortestosterone derivatives: high-affinity ligands and potent partial agonists of the androgen receptor. J Med Chem *47*, 4985-4988.
2. Pindur, U., and Schall, T. (1994). Proton Acid-Catalysed Transformations of Estrogen Derivatives: New Results and Some Mechanistic Aspects of the Kober Colour Reaction. Arch Pharm *327*, 637-642.
3. Lin, X., Hou, C., Li, H., and Weng, Z. (2016). Decarboxylative Trifluoromethylating Reagent [Cu(O2 CCF3)(phen)] and Difluorocarbene Precursor [Cu(phen)2][O2 CCF2 Cl]. Chemistry *22*, 2075-2084.
4. Triggle, D.J., Ridley, H.F., and DeMaio, D.M. (1969). Some 3-alkoxyestra-1,3,5(10)-trien-17-beta-ols. J Med Chem *12*, 346.
5. Tedesco, R., Thomas, J.A., Katzenellenbogen, B.S., and Katzenellenbogen, J.A. (2001). The estrogen receptor: a structure-based approach to the design of new specific hormone–receptor combinations. Chem Biol *8*, 277-287.
6. Santra, S., and Guin, J. (2015). Enhanced Reactivity of Aerobic Diimide Olefin Hydrogenation with Arylboronic Compounds: An Efficient One-Pot Reduction/Oxidation Protocol. Eur J Org Chem *2015*, 7253-7257.
7. Hostetler, E.D., Jonson, S.D., Welch, M.J., and Katzenellenbogen, J.A. (1999). Synthesis of 2-[(18)F]Fluoroestradiol, a Potential Diagnostic Imaging Agent for Breast Cancer: Strategies to Achieve Nucleophilic Substitution of an Electron-Rich Aromatic Ring with [(18)F]F(-). J Org Chem *64*, 178-185.
8. Shi, Y., and Koh, J.T. (2002). Functionally orthogonal ligand-receptor pairs for the selective regulation of gene expression generated by manipulation of charged residues at the ligand-receptor interface of ER alpha and ER beta. J Am Chem Soc *124*, 6921-6928.
